# Mammalian embryo comparison identifies novel pluripotency genes associated with the naïve or primed state

**DOI:** 10.1242/bio.033282

**Published:** 2018-07-19

**Authors:** Andreia S. Bernardo, Alice Jouneau, Hendrik Marks, Philip Kensche, Julianna Kobolak, Kristine Freude, Vanessa Hall, Anita Feher, Zsuzsanna Polgar, Chiara Sartori, Istvan Bock, Claire Louet, Tiago Faial, Hindrik H. D. Kerstens, Camille Bouissou, Gregory Parsonage, Kaveh Mashayekhi, James C. Smith, Giovanna Lazzari, Poul Hyttel, Hendrik G. Stunnenberg, Martijn Huynen, Roger A. Pedersen, Andras Dinnyes

**Affiliations:** 1 The Anne McLaren Laboratory for Regenerative Medicine, University of Cambridge, Cambridge CB2 0SZ, UK; 2 Developmental Biology Department, The Francis Crick Institute, 1 Midland Rd, Kings Cross, London NW1 1AT, UK; 3 UMR BDR, INRA, ENVA, Université Paris Saclay, 78350, Jouy en Josas, France; 4 Center for Molecular and Biomolecular Informatics, Radboud Institute of Molecular Life Sciences, Radboud University Medical Centre, PO Box 9101, 6500 HB Nijmegen, The Netherlands; 5 Department of Molecular Biology, Faculty of Science, Radboud University, Radboud Institute for Molecular Life Sciences (RIMLS), 6500 HB Nijmegen, The Netherlands; 6 BioTalentum Ltd, Gödöllő, 2100 Godollo, Hungary; 7 Department of Veterinary Clinical and Animal Sciences, Faculty of Health and Medical Sciences, University of Copenhagen, Groennegaardsvej 7, 1870 Frederiksberg C, Denmark; 8 Avantea, Laboratory of Reproductive Technologies, Cremona, 26100 Cremona, Italy; 9 Department of Physiology, University of Lausanne, CH-1005 Lausanne, Switzerland; 10 Molecular Animal Biotechnology Laboratory, Szent István University, H-2100 Godollo, Gödöllő, Hungary; 11 Departments of Equine Sciences and Farm Animal Health, Faculty of Veterinary Medicine, Utrecht University, 3584CL Utrecht, The Netherlands

**Keywords:** Dusp6, Gjb5, Trip6, Naïve, Pluripotency, Primed

## Abstract

During early mammalian development, transient pools of pluripotent cells emerge that can be immortalised upon stem cell derivation. The pluripotent state, ‘naïve’ or ‘primed’, depends on the embryonic stage and derivation conditions used. Here we analyse the temporal gene expression patterns of mouse, cattle and porcine embryos at stages that harbour different types of pluripotent cells. We document conserved and divergent traits in gene expression, and identify predictor genes shared across the species that are associated with pluripotent states *in vivo* and *in vitro*. Amongst these are the pluripotency-linked genes *Klf4* and *Lin28b*. The novel genes discovered include naïve- (*Spic, Scpep1* and *Gjb5*) and primed-associated (*Sema6a* and *Jakmip2*) genes as well as naïve to primed transition genes (*Dusp6* and *Trip6*). Both *Gjb5* and *Dusp6* play a role in pluripotency since their knockdown results in differentiation and downregulation of key pluripotency genes. Our interspecies comparison revealed new insights of pluripotency, pluripotent stem cell identity and a new molecular criterion for distinguishing between pluripotent states in various species, including human.

## Abstract (precis)

**Summary:** Interspecies comparison of mouse, bovine and pig embryos revealed conserved genes which distinguish between naïve and primed pluripotency states, including in human. Some of these genes interfere with the pluripotency network and lead to differentiation.

## INTRODUCTION

Understanding early embryonic development, particularly pluripotency, is paramount for establishing pluripotent stem cell (PSC) models. These models are powerful tools for *in vitro* disease modelling as well as for improving the health of domestic species (e.g. cattle and pig) and for the production of livestock with specific traits through genetic engineering. For practical and ethical reasons, most mammalian development studies have used the mouse as the model organism. However, early mouse development diverges from that of primates and of domestic species.

The blastocyst stage is the first key embryonic hallmark of development and mammalian embryos at this stage look relatively similar, i.e. embryos have an outermost layer, the trophectoderm, and an inner layer of cells, the inner cell mass (ICM). The ICM includes the cells that will form the primitive endoderm and the pluripotent epiblast cells (Cockburn and Rossant, 2010). Notably, mouse species are among the few mammals that can halt their development and enter diapause at this stage (Renfree and Fenelon, 2017). During the next several days, the shape and size of the embryo changes dramatically. The epiblast cells self-organise into an epithelium shaped as a cup in rodents, or as a flat disc in primates as well as in the pig and bovine (ungulate species). At this stage, the embryo is composed of an epithelial radially symmetric epiblast (ERSE) which is surrounded by the extraembryonic endoderm. As gastrulation proceeds, epiblast cells undergo epithelial to mesenchymal transition and migrate to form the definitive endoderm and mesoderm germ layers. This migration causes the embryo to elongate, generating an asymmetrical elongated anterior to posterior epiblast (APE) (Degrelle et al., 2005; Lawson et al., 1991; Maddox-Hyttel et al., 2003). The epiblast in ERSE and APE stages remain pluripotent as suggested by clonal studies performed in the mouse (Lawson et al., 1991) and by chimera studies using mouse epiblast stem cells (mEpiSCs) (Huang et al., 2012; Masaki et al., 2016). Despite many conserved morphological features, the establishment of PSC cultures *in vitro* has proved challenging in species other than mouse and primates.

Human and mouse pluripotent stem cells exist in two different states that have been termed ‘naïve’ and ‘primed’ (Nichols and Smith, 2009). mEpiSCs, conventional human embryonic stem cells (hESCs) and induced PSCs (human-iPSCs), represent the primed state and correspond to the post-implantation epiblast (Ng and Surani, 2011; Chen et al., 2016). In contrast, mouse embryonic stem cells (mESCs) and induced PSCs (mouse-iPSCs) represent the naïve state and correspond to the E4.5 epiblast despite being derived from ICM epiblast cells (Boroviak et al., 2014). Different methods have been proposed to revert hESCs to a naïve state (Chan et al., 2013; Gafni et al., 2013; Theunissen et al., 2014; Takashima et al., 2014) or to derive naïve ESCs from human ICM cells (Guo et al., 2016). However, only some of these naïve hPSCs have been shown to correlate with the monkey pre-implantation epiblast stage (Nakamura et al., 2016).

mESCs and mEpiSCs have different culture requirements, express specific gene sets and have different abilities to form chimeras (Huang et al., 2012; Nichols and Smith, 2009; Pauklin et al., 2011). These differences have been determined by comparative studies performed between mESCs and mEpiSCs and further substantiated by in-depth analysis of mouse embryonic development (Boroviak et al., 2014). Similar analysis of marmoset and cynomolgus monkey embryo development revealed striking differences between these mammalian species and the mouse (Boroviak et al., 2014; Nakamura et al., 2016). However, little is known about the mechanisms underlying pluripotency *in vivo* during early embryonic development of ungulate species from which stem cell derivation has not been successfully achieved, except for an isolated report on the derivation of pig mEpiSCs which has not been validated by other labs (Alberio et al., 2010).

Molecular comparisons of pluripotent embryonic tissues between species could provide valuable insights into disparities in the efficacy of pluripotent stem cell derivation and the nature of the stem cells derived. To this end, we compared the transcriptomes of mouse (*Mus musculus*), pig (*Sus scrofa*) and bovine (*Bos taurus*) ICM, ERSE and APE stage-matched embryos devoid of their extraembryonic tissues. By focusing on gene orthologues across the three species, we identified conserved and non-conserved gene expression patterns between the mouse and the ungulate species. We then intersected the set of conserved genes that were differentially expressed between *in vivo* stages with those that characterised the naïve or primed state found in mouse pluripotent cells *in vitro*. This allowed us to: (1) identify a set of novel predictor genes, important for the identity and/or maintenance of the ‘naïve’ state or the ‘primed’ state and (2) understand the evolutionary conservation of the naïve and primed pluripotency circuitry.

## RESULTS

### Comparison of mouse, pig and bovine embryos reveals different paces of development in these species

Pluripotent cells are present within the ICM, giving rise to the early epiblast, and in the late post-implantation epiblast of the ERSE and APE stages. To characterise these stages of pluripotency at a molecular level we set out to compare the transcriptomes of mouse and ungulate embryos.

Interspecies comparison studies depend on reliable stage matching of embryos. To ensure the comparison of equivalent embryonic stages, we first performed *in situ* hybridisation with *Oct4 (Pou5f1)*, *Nanog* and *Sox2* probes in E3.5 (ICM stage), E6.25 (ERSE stage) and E7.25 (APE stage) mouse embryos and in a range of pig and bovine embryos (E4.0-E7.0 to align the ICM stage; E7.0-E16.0 to align the ERSE stage; E12.5-E18.0 to align the APE stage). Nodal was also used as it shows a dynamic expression during epiblast development both in mouse and bovine embryos (Varlet et al., 1997; van Leeuwen et al., 2014). Finally, we studied *Brachyury* (*T*) expression in APE stage embryos to detect the appearance of the primitive streak.

**Fig. 1.**
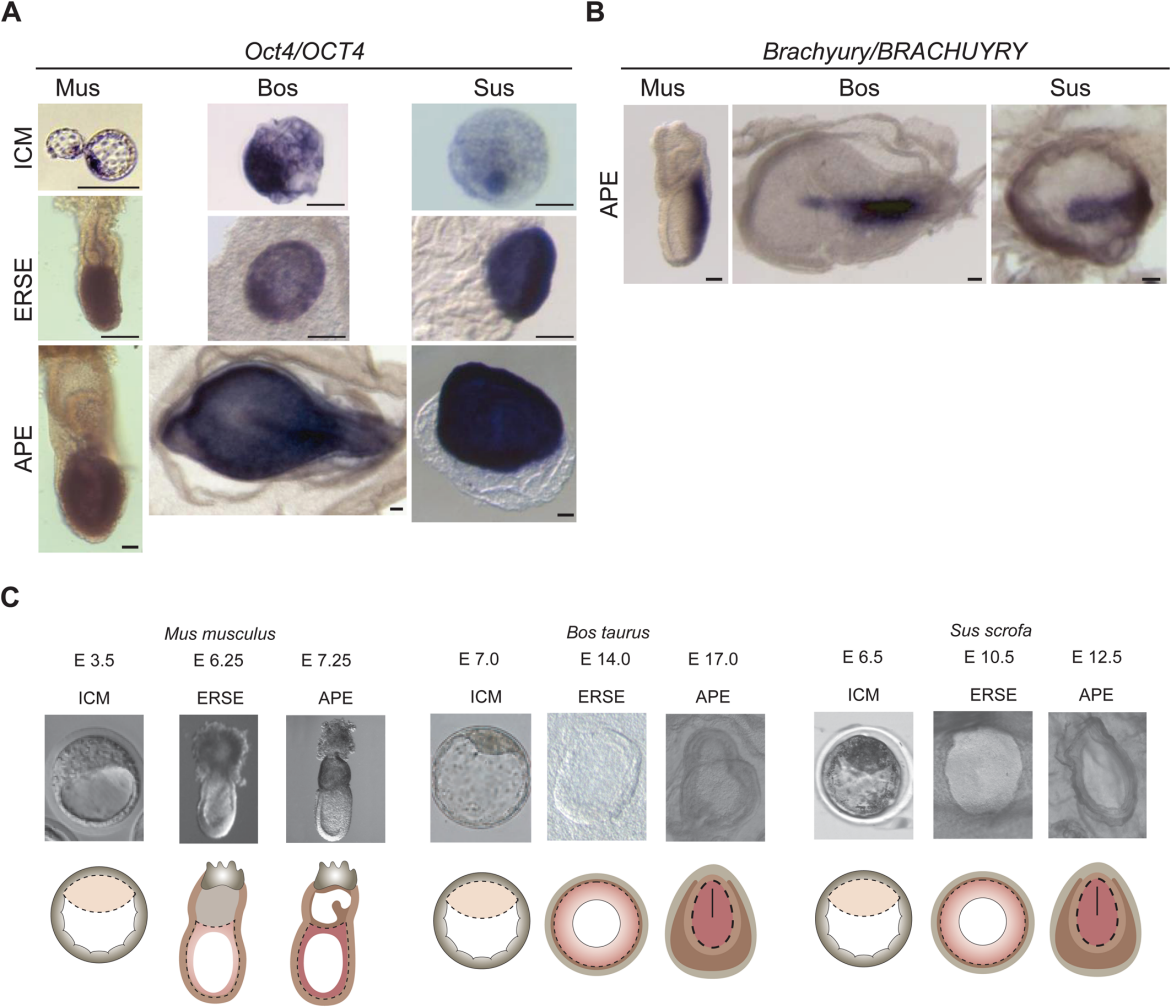
**Alignment of embryo stages across species.** (A) *In situ* hybridisation for Oct4/OCT4 in ICM, ERSE and APE stage embryos. Two to six embryos were analysed per species and these are representative embryos. Scale bars: 100 µm. (B) *In situ* hybridisation for Brachyury/BRACHYURY in APE stage embryos. Two to six embryos were analysed per species and these are representative embryos. Scale bars: 200 µm. (C) Illustration of the samples dissected for RNA-sequencing. The epiblast is depicted in shades of pink; trophectoderm in grey; extraembryonic endoderm in light brown and extraembryonic mesoderm in dark brown. The line in the APE stages represents the primitive streak. Dashed lines show epiblast cells dissected for total RNA isolation.

Based on *Oct4*/*OCT4*, *Nanog/NANOG* and *Sox2/SOX2* expression in the inner layer of the newly formed blastocyst, we found that the stage best corresponding to mouse E3.5 was at E6.5/7 in both pig and cattle. The equivalent of the mouse ERSE stage was at E10.5 in pig and E14.0 in cattle, with *OCT4* expression demarcating a near perfect radially symmetrical epiblast, and *NANOG*, *SOX2* and *NODAL* expression demarcating the entire epiblast but showing an asymmetric distribution within it as previously described for the mouse [Fig. 1A; Fig. S1A-C (Varlet et al., 1997; Avilion et al., 2003; Chambers et al., 2003)]. Finally, we identified a characteristic asymmetric line of *BRACHYURY* expression defining the APE stage of pig and bovine embryos at E12.5 and E17.0, respectively (Fig. 1B), while *OCT4*, *NANOG*, *SOX2* and *NODAL* were still well expressed in the epiblast (Fig. 1A; Fig. S1A-C).

These data defined the stages at which we compared the transcriptome of ungulate embryos with the mouse ICM, ERSE and APE stages (Fig. 1C).

### Mouse, pig and bovine transcriptional landscapes of the early and late epiblast cluster together

**Fig. 2.**
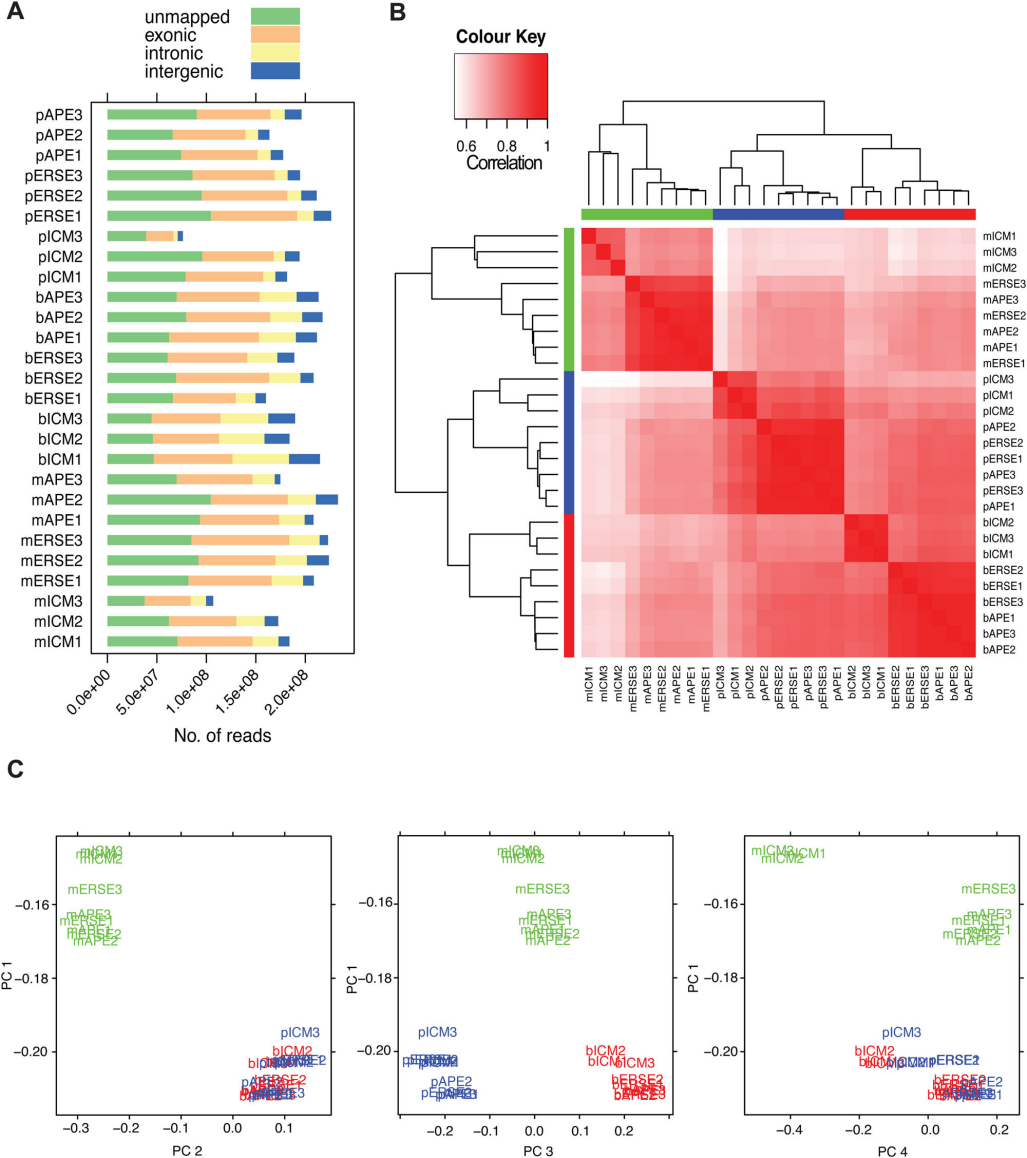
**Transcriptional landscapes distinguish ICM from the late epiblast in both mice and ungulate species.** (A) Total number of reads not mapped uniquely and reads mapping uniquely to exonic, intronic, or intergenic regions. (B) Heatmap of Pearson correlations coefficients. The dendrograms on the margins represent the average linkage clustering of the samples. (C) Projection of samples according to their principal components analysis. PC1, 71% of variance explained; PC2, 16%; PC3, 4%; PC4, 2%.

Because of the small RNA content of mammalian early embryos, we first assessed different RNA amplification methods (Fig. S2A-D). This analysis showed that the SMARTer method was superior to the other methods tested. Next, ICM, ERSE and APE stage embryos were dissected to isolate the pluripotent cell populations, RNA was purified and libraries were prepared. Transcriptome-wide profiling of the libraries resulted in 76–233 million reads per sample, yielding 37–168 million uniquely mappable reads (Fig. 2A; Table S1). These were used for transcript quantification (Fig. S2E) and 3′ bias assessment (Fig. S2F). Good coverage across the complete length of transcripts was observed. As expected, most of the reads fell into exons (Fig. 2A). Matched samples correlated well with each other (Fig. 2B), however, sample pICM3 was of poor quality [Fig. S2E and cluster analysis based on orthologue genes (data not shown)] and was therefore omitted from differential expression analyses. The purity of our dissected samples was confirmed by: (1) the low/absence of trophoblast-associated gene expression in our samples and (2) the low/absence of extraembryonic ectoderm- and endoderm-associated gene expression in the ERSE samples (data not shown).

The DNA sequence similarity between bovine, porcine and mouse varies. To avoid having to discard read sequence alignments in order to compare data across species, our analysis focused on orthologous genes common to the three species (Table S2). In all, 11,444 1:1:1 orthologous genes (i.e. genes that have only one counterpart in the studied species) were identified in our dataset. By clustering the samples according to their transcriptome we found that: (1) the samples formed three distinct clusters according to the species; (2) ungulate samples clustered away from mouse samples; and (3) the sample groups (per embryonic stage) could be split into ICM and late epiblast (ERSE and APE) (Fig. 2B). This observation was further confirmed by principal component analysis, in which the differences between ERSE and APE samples were too small to define distinct clusters (Fig. 2C), and is in keeping with what has been observed in the monkey for similar staged embryos (Nakamura et al., 2016). This analysis also highlighted that in the mouse the difference between ICM and ERSE/APE was bigger than in bovine or pig (Fig. 2C). In conclusion, our transcriptome-wide profiling has yielded a robust dataset for performing inter-species comparisons.

### Significant inter-species differences in core pluripotency genes

**Fig. 3.**
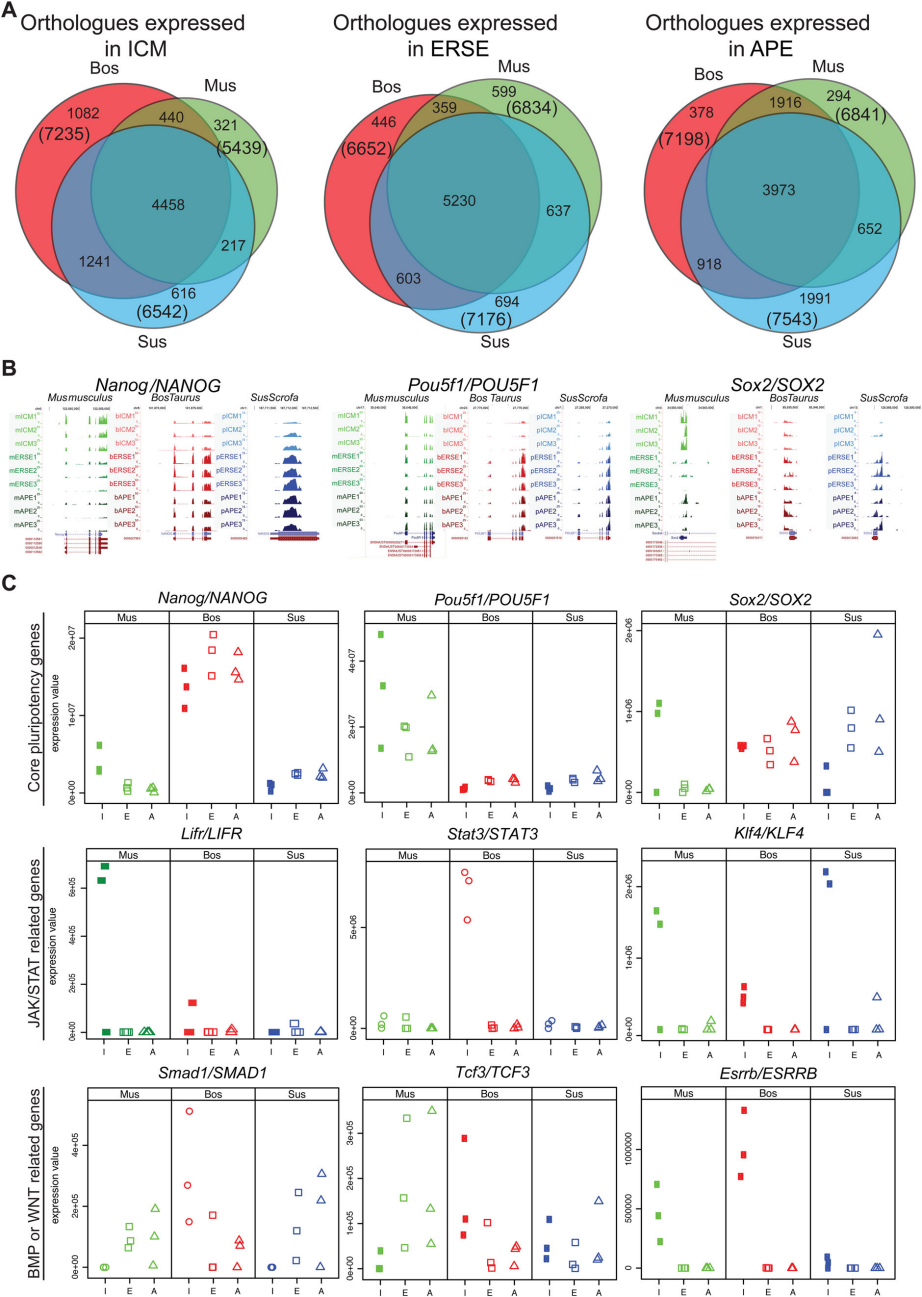
**Significant inter-species differences in core pluripotency genes were detected.** (A) Venn diagrams showing the overlap between expressed orthologues at each developmental stage in the three species: mouse (green), cattle (red) and pig (blue). (B) UCSC browser views of the RNA-seq coverage plot of uniquely mapped reads for *Pou5f1*/*POU5F1*, *Nanog/NANOG* and *Sox2/SOX2* genes in the different species and at the different stages. (C) Dot plots showing the values of expression from the RNA-seq data of known pluripotency associated genes. I, ICM; E, ERSE; A, APE.

To evaluate the conservation and divergence in gene expression of the studied species we compared the orthologues expressed at each embryonic stage (Fig. 3A). Between 5439 and 7543 orthologous genes were expressed by each species at a given stage. A large proportion of the stage-specific genes were commonly expressed across species (up to 82% of the genes expressed by each species). Analysis of the commonly expressed genes at each embryonic stage using the Kyoto Encyclopaedia of Genes and Genomes (KEGG) Pathway Database only revealed an enrichment of genes involved in metabolism and energy, cell cycle and DNA repair (Table S3A).

*Oct4* (*Pou5f1*), *Sox2* and *Nanog* are considered the core pluripotency triad in human and mouse pluripotent cells. They also play essential roles during early development in the mouse embryo (Osorno and Chambers, 2011). Strikingly, the expression values extracted from our dataset revealed that the expression of *OCT4*, *NANOG* and *SOX2* tended to be upregulated between ICM to ERSE in ungulates, while being decreased (*Nanog* and *Sox2*) or stable (*Oct4*) in mouse (Fig. 3B,C). This was confirmed by *in situ* hybridisation, which also denoted a low level of *NANOG* and *SOX2* in ungulate ICM (Fig. 1A; Fig. S1A,B). These differences highlight nuances in gene expression, which might reflect a divergence in the mechanisms of pluripotency maintenance between mouse and ungulate species.

To get further insight into the mouse and ungulate pluripotency circuitry we searched our ICM and ERSE datasets for genes known to be involved in pluripotency (Fig. 3C; Fig. S3A and Table S3B). In the mouse, naïve pluripotency can be maintained with LIF and BMP signalling (Qi et al., 2004). Thus, it was surprising that the expression of the BMP effector *Smad1* was hardly detected in mouse and pig ICM but was present in ERSE embryos. *LIFR* expression, a gene upstream of the JAK/STAT pathway, was lacking in both the bovine and porcine ICM (Fig. 3C; Fig. S3A in yellow), suggesting that ungulate naïve pluripotency does not rely on LIF signalling and *STAT3* is likely activated through an alternate receptor or pathway. Interleukins 6 and 11 are possible ligand candidates since we detect expression of their receptors in both bovine and porcine embryos. Klf4/KLF4 expression at the ICM of all species, which in mESCs is regulated by Stat3 following activation by LIF (Bourillot et al., 2009), further suggests that in ungulates the JAK/STAT pathway is also activated to maintain pluripotency. Naïve pluripotency in the mouse can also be maintained independently of Jak/Stat3 and Bmp/Smad1 signalling through the pharmacological inhibition of both Gsk3β (which mimics Wnt signalling) and FGF signalling (Ying et al., 2008). Gsk3β has many targets, including Esrrb, which is one of the main effectors through which the Gsk3/β-catenin/Tcf3 axis modulates ESC self-renewal (Martello et al., 2012). We found that *Esrrb/ESRRB* was expressed in the ICM stage of all species, although at a considerable lower level in pig (Fig. 3C; Table S3B) and was downregulated in ERSE. However, the terminal target of the Wnt pathway, *Tcf3,* was hardly detected in the mouse ICM and its expression was upregulated in ERSE stage embryos, while in ungulates, *TCF3* was only (lowly) expressed at the ICM stage (Fig. 3C; Fig. S3A and Table S3B).

Altogether, our data identified conservation traits across species in terms of the transcriptional machineries, and divergences in the timing, level and/or pattern of gene expression. Moreover, we have shown that there are more similarities within the ungulate embryos than there are between ungulate and murine embryos in terms of stage-specific gene expression, transcript localisation in the embryo and core pluripotency gene expression. The identified differences from mouse pluripotent embryonic tissues likely explain, at least in part, why it has been inefficient to derive pluripotent stem cells from ungulate species using approaches suitable for mouse stem cell derivation.

### Differential expression analysis identifies common stage-specific pluripotency-associated genes

**Fig. 4.**
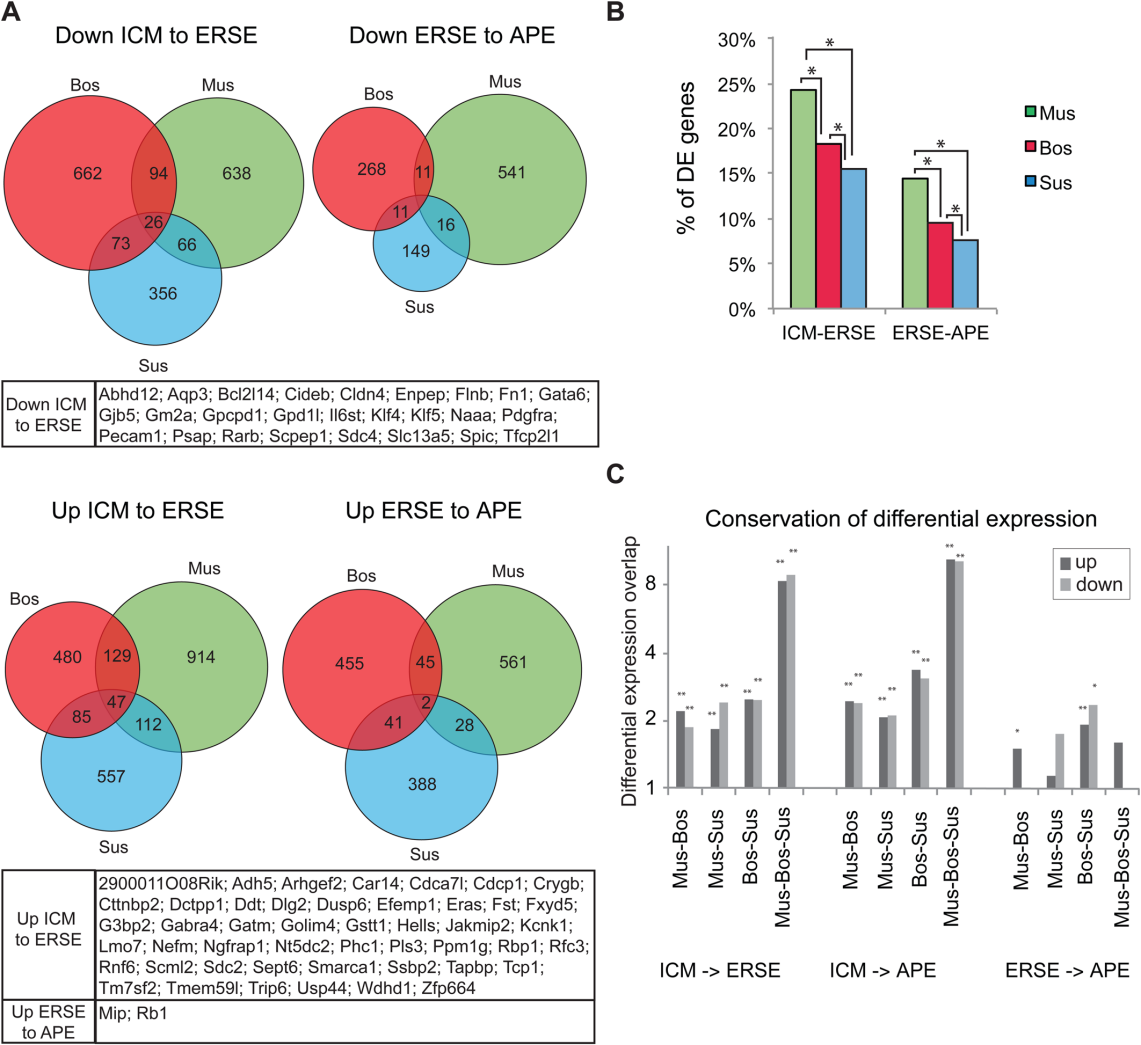
**Differential expression analysis identifies common stage specific pluripotency-associated genes.** (A) Venn diagrams showing the intersections of differentially expressed genes during epiblast development in the three species (with q-value ≤0.25, minimum fold change 1.5). Below each Venn is shown the list of genes belonging to the central territory of the diagram. (B) Graphs showing the proportion of differentially expressed (DE) 1:1:1 orthologues across stages. *P<10-17, χ^2^ test. (C) Graphs showing the conservation of differential expression between species. The conservation is expressed as the number of genes that are differentially expressed between stages in two or in three species, divided by the expected overlap if the same number of genes were independently sampled from the 1-1-1 orthologues in those genomes. *P*-values are based on the hypergeometric distribution, **P<1E -4, *P<0.01.

To further assess the degree of conservation between species, we compared the 1:1:1 orthologues that are differentially expressed between stages in each species (Table S4A). Each stage transition category was plotted as a Venn diagram showing the extent of intersection between species (Fig. 4A; Table S4B). Several hundred genes were differentially regulated between stages in each species (with the mouse having the highest percentage of differentially expressed genes, Fig. 4B). However, the overlap of differential expression between the species was quite small (Fig. 4A). In particular, few genes were jointly up or downregulated between the ERSE and APE stage, probably reflecting the similarity between these stages (Fig. 2C). Nonetheless, between two species the number of genes in the overlap for the ICM to ERSE transition was about twice what would be expected from a random occurrence (*P*<10^−4^, hypergeometric test; Fig. 4C). The overlap between the three species was even more substantial; about eight times what was expected had it been random (*P*<10^−4^, hypergeometric test; Fig. 4C). Overall, the overlaps for ICM to ERSE and ICM to APE were highly significant.

We noted that most changes in gene expression between sequential stages occur during the ICM to ERSE transition and the conserved changes included the downregulation of a number of pluripotency-associated genes whose expression is lower in mEpiSCs than in mESCs, including *Klf4/KLF4*, *Klf5/KLF5* and *Pecam1/PECAM1* (Fig. 4A). *Tfcp2l1*, a target of Stat3 that promotes self-renewal in mESCs (Ye et al., 2013), and *Spic,* a member of the Ets-family expressed and required at the ICM stage in the mouse (Kageyama et al., 2006; Pelton et al., 2002), were also downregulated during the ICM-ERSE transition in all three species (Fig. 4A). The naïve pluripotency gene Tbx3, of which expression is downregulated between mESCs and mEpiSCs (Russell et al., 2015), was also downregulated during the ICM-ERSE transition, albeit in mouse the downregulation was not significant (Table S4). Moreover, some known mouse hypoblast markers including *Scd4/SCD4*, *Gata6/GATA6*, and *Pdgfra/PDGFRa* (Artus et al., 2010; Chazaud et al., 2006; Kurimoto et al., 2006) were also downregulated across the three species during the ICM-ERSE transition (Fig. 4A). Conversely, other pluripotency associated genes such as *Eras/ERAS* (Takahashi et al., 2003) and *Lin28b/LIN28B* (Darr and Benvenisty, 2009) were upregulated in ERSE or APE stage embryos, respectively (Fig. 4A; Fig. S4, respectively).

Together, our analysis identified a small set of genes whose differential expression is conserved within the three species.

### Comparison of *in vivo* and *in vitro* transcriptomes unveils novel stage-specific pluripotency-associated genes

Many developmentally important genes are conserved throughout evolution and here we have found that the expression of a small pool of highly conserved genes characteristically changes during the ICM to late epiblast (ERSE and APE) transition. As some of these genes were not previously recognised as being associated with pluripotency, we asked whether they are also differentially expressed in the naïve and primed pluripotency states of mouse pluripotent cell lines. To this end, we compared our RNA-sequencing dataset with published data for mESCs (Marks et al., 2012) and mEpiSCs (Veillard et al., 2014). We used data for mESCs grown in serum+Lif (BMP dependent), or in 2i+Lif (ERK/GSK3 inhibition dependent), where cells remain in a naïve state of pluripotency.

Correlation analysis of mouse *in vivo* and *in vitro* samples showed that the ICM stage did not correlate well with any pluripotent cells whereas both ERSE and APE had some similarities with serum-mESCs and mEpiSCs (Fig. S5A). Principal component analysis performed using the three species samples further confirmed these findings (Fig. S5B); i.e. by plotting the samples on PC5, which separates the samples according to their developmental position. It is clear that while ERSE/APE stage embryos group well with late epiblast derived stem cells (mEpiSCs), ICM stage embryos do not group with any of the ICM-derived stem cells (2i-mESCs or serum-mESCs), although they are slightly closer to 2i-mESCs (Fig. S5B). This is in keeping with previous findings that show that naïve mESCs resemble the pre-implantation E4.5 mouse epiblast rather than the ICM (Chen et al., 2016; Boroviak et al., 2014), while mEpiSCs resemble the post-implantation epiblast (Chen et al., 2016; Kojima et al., 2014). Note that PC5 only explains 3% of the variation in the data, while PC1-PC4 correspond to species differences as well as *in vivo*/*in vitro* differences and do not explain the biology of the samples.

**Fig. 5.**
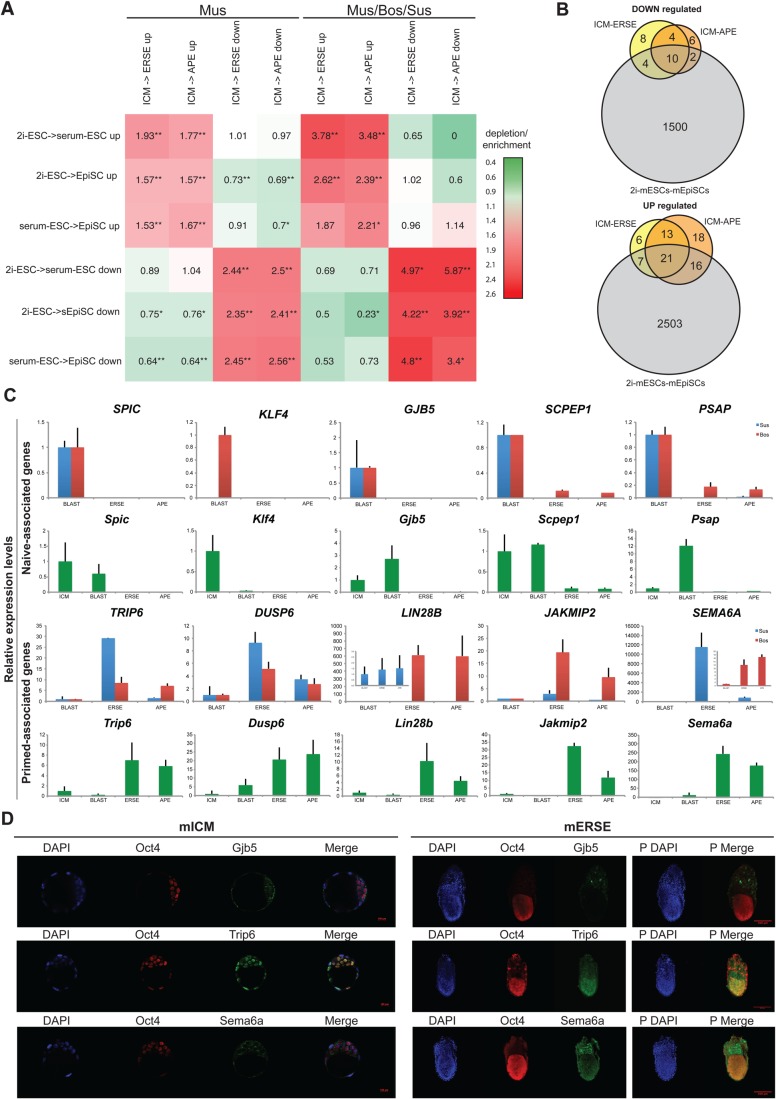
***In vivo/in vitro* comparison underpinned relevant naïve and primed pluripotency-associated genes.** (A) Heatmap of the conservation of differentially expressed genes between *in vivo* and *in vitro* samples. The overlap is expressed for genes that are differentially expressed in mouse (left) and for genes whose differential expression is conserved among the three species (right). Log-fold changes are shown with respect to a randomised control, as in Fig. 4C. *P*-values are based on the hypergeometric distribution, **P<1E-4, *P<0.01. (B) Venn diagrams showing the intersections between the sets of differentially expressed genes in 2i-mESCs versus mEpiSCs and our dataset of common differentially expressed genes across species (ICM versus ERSE or APE). (C) RT-qPCR showing the expression of some of the newly discovered pluripotency-associated genes in bovine, porcine and mouse embryos: isolated ICM (for mouse only), whole blastocyst, ERSE and APE. Expression values are normalised against *Gapdh (*blue, Sus; red, Bos; green, Mus). Three independent biological replicates were analysed. Error bars represent the standard deviation. (D) Immunostaining of Gjb5, Trip6 and Sema6a together with Oct4 in mouse blastocysts and ERSE stage embryos. DAPI staining is shown in blue. Right panels indexed with a ‘P’ represent the maximum projection of the z-stacks for the ERSE embryos. Three to four independent embryos were analysed and these are representative embryos.

To investigate how the mouse PSC lines compare with their *in vivo* equivalents, we determined the changes occurring during the 2i-ESC to serum-ESC to mEpiSC transitions and cross-compared this datasets with our previous *in vivo* analysis (Table S5A). The overlap of differentially expressed genes between *in vivo* and *in vitro* mouse samples suggests that the 2i- to serum-mESC transition reflects the ICM-ERSE transition better than the serum-mESCs to mEpiSCs transition, especially for the upregulated genes (Fig. 5A). This is even clearer when the *in vivo*/*in vitro* comparison is done with the pool of genes that are conserved across species (Fig. 5A, right), highlighting the relevance of the conserved genes for pluripotency. The intersection of stage-specific conserved genes with the *in vitro* samples further showed that only a few genes are up or downregulated both *in vivo* and *in vitro* (Fig. 5B; Fig. S5C and Table S5B). These intersections also highlight that some mEpiSC-associated genes are already upregulated in serum-mESCs (29 out of the 44 genes upregulated between 2i-mESCs and mEpiSCs are also upregulated between 2i- and serum-mESCs, Table S5B) while various 2i-mESC-associated genes are significantly decreased in serum-mESCs (10 out of the 16 genes downregulated between 2i-mESCs and mEpiSCs are already downregulated between 2i- and serum-mESCs, Table S5B). As such, serum-mESCs can be considered as a transitional stage between naïve and primed pluripotency, which is in keeping with what has recently been reported (Chen et al., 2016).

Together these analyses revealed that despite the differences between ICM and ICM-derived cell lines, the transition between early and late epiblast *in vivo* is recapitulated *in vitro* and reflected by a small group of conserved genes. Surprisingly, only a few of these have been previously associated with pluripotency.

### Validation of newly discovered genes’ expression *in vivo*

It is likely that the genes which show significant changes in expression both *in vitro* (in mouse pluripotent cells) and *in vivo* (in the three species) have important conserved roles in the modulation of pluripotency. To validate whether these genes are indeed expressed *in vitro* and *in vivo* we selected a set of genes associated with either the naïve/ICM state (*Klf4/KLF4, Gjb5/GJB5, Spic/SPIC, Scpep1/SCPEP1* and *Psap/PSAP*) or the primed/ERSE/APE state (*Trip6/TRIP6, Dusp6/DUSP6, Jakmip2/JAKMIP2, Lin28b/LIN28b*, *Lpar4/LPAR4*, *Car14/CAR14* and *Fst/FST*). We further included *Sema6A*/*SEMA6A*, which was upregulated in the 2i-mESC to mEpiSC transition as well as in the ICM to ERSE transition in both cow and pig but not mouse, as *SEMA6A* has been shown to be important for self-renewal in hESCs (Dowell et al., 2014). The expression levels of the selected genes in our sequenced *in vivo* data are shown in Figs S5D and S6A.

We first validated the expression of these genes in embryonic tissues by analysing blastocyst (ICM stage) and dissected epiblasts at ERSE and APE stage embryos of the three species. Real-time qPCR analysis of murine, bovine and porcine embryos revealed that the levels of Spic/SPIC, *Gjb5/GJB5, Scpep1/SCPEP1* and *Psap*/*PSAP* expression were significantly higher in ICM stage embryos than in ERSE or APE stage embryos (Fig. 5C). The same was true for *Klf4/KLF4* in the mouse and bovine but unexpectedly, this gene was not detected in porcine embryos despite its presence in the associated sequencing data (Figs 5C, 3C). The opposite trend, i.e. higher expression in ERSE/APE, was confirmed for *Trip6/TRIP6*, *Dusp6/DUSP6, Lin28b/LIN28B, Jakmip2/JAKMIP2*, and *Sema6a/SEMA6A* expression (Fig. 5C) as well as for *Lpar4*, *Car14* and *Fst* in the mouse (Fig. S5E). Immunostaining was then performed for Klf4, Gjb5, Trip6 and Sema6A on mouse embryos at the ICM and ERSE stage. Klf4 and Trip6 are nuclear proteins, whereas Gjb5 and Sema6A are trans-membrane proteins. ICM cells clearly expressed both Gjb5 and Klf4, whereas these proteins became undetectable in the epiblast at the ERSE stage (Fig. 5D; Fig. S5F). Conversely, Sema6A expression was weak in the ICM cells, and high in the ERSE stage epiblast (Fig. 5D). The only discrepancy with the RNA-Seq data regarded Trip6 expression, which was present at seemingly similar levels at both stages (Fig. 5D), unlike what we observed by QPCR (Fig. 5C).

In summary, we largely confirmed *in vivo* the expression trends highlighted by our transcriptome analysis for the sets of genes analysed. In most cases, we also detected the corresponding change at protein level. Thus, our interspecies approach highlighted embryonic stage specific characteristics not previously known.

### Newly discovered genes can be used as predictors of the naïve/primed pluripotency state

We next asked if these genes could be used as ‘predictors’ of the naïve/primed pluripotency state *in vitro* or as mESC ‘differentiator’ genes, i.e. genes which could distinguish between ground state 2i-mESCs and serum-mESCs.

**Fig. 6.**
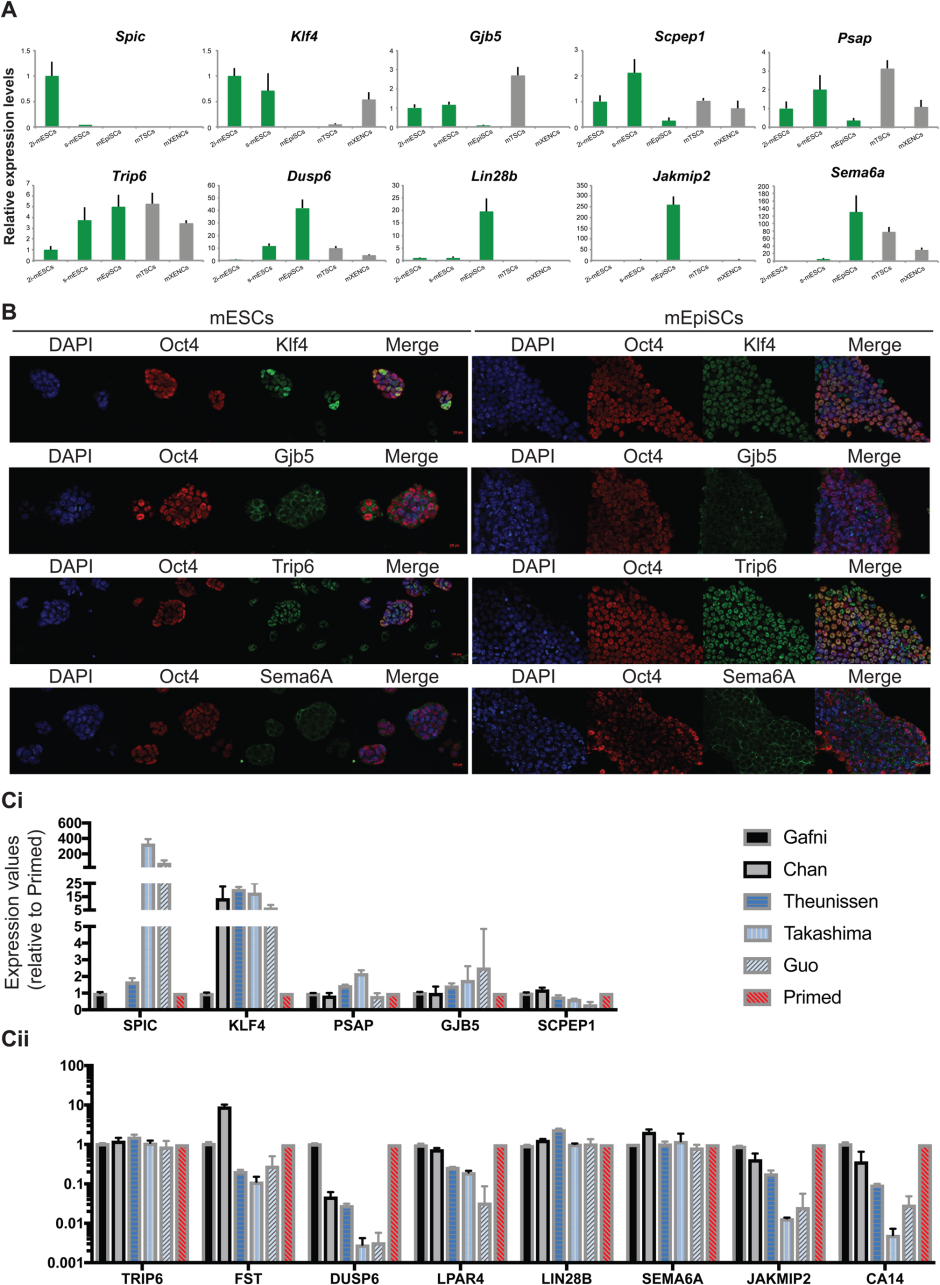
**Common *in vivo* naïve and primed pluripotency-associated genes also distinguish naïve and primed human stem cells.** (A) RT-qPCR showing the expression levels of newly discovered pluripotency-associated genes in mouse stem cells (2i-mESCs or serum-mESCs; mEpiSCs; mTSCs; mXENCs). Expression values are normalised against *Gapdh*. Three independent biological replicates were analysed. Error bars represent the standard deviation. (B) Immunostaining of Klf4, Gjb5, Trip6 and Sema6A together with Oct4 in 2i-mESCs and mEpiSCs. DAPI staining is shown in blue. These are representative images of stained cultures. (C) Expression of the newly discovered pluripotency-associated genes in human conventional (primed) ESCs, human ‘reset’ (naïve) ESCs and human embryo-derived naïve ESCs. Data were extracted from Gafni et al. (2013), Chan et al. (2013), Theunissen et al. (2014), Takashima et al. (2014) and Guo et al. (2016). (i) Genes associated with naïve-pluripotency. (ii) Genes associated with primed-pluripotency, including the transition genes. Expression values are presented in Log10 scale.

Real-time qPCR was performed in mESCs (grown in 2i or serum), mEpiSCs and, for comparative purposes, in other embryo-derived stem cell lines, i.e. trophoblast (mTSCs) and primitive endoderm (mXENCs) stem cells (Fig. 6A; Fig. S6B). As predicted, *Klf4, Gjb5*, *Scpep1* and *Psap* were expressed at significantly higher levels in mESCs than in mEpiSCs and were thus considered as predictors of the naïve state. These genes were also expressed at considerably high levels in mTSCs and/or mXENCs. Interestingly, *Spic* was the only gene which expression was restricted to 2i-mESCs, hence a new marker of ground state of pluripotency. Conversely, *Lin28b, Sema6a, Jakmip2* and *Car14* were highly expressed in mEpiSCs compared to mESCs. Expression of *Dusp6* (Fig. 6A), *Fst* and *Lpar4* (Fig. S6B) was strongly upregulated in serum-mESCs and in EpiSCs (*Fst*) or even more upregulated in EpiSCs (*Dusp6* and *Lpar4*). Low expression of these genes could therefore be considered as a feature of the naïve state. Lastly, *Trip6* expression was detected in all stem cells, with only a modest upregulation from 2i-mESCs to serum-mESCs and mEpiSCs.

Immunostaining analysis (Fig. 6B) further confirmed that both Gjb5 and Klf4 were expressed in mESCs and their expression was downregulated in mEpiSCs. Conversely, Sema6A expression was upregulated in mEpiSCs. Trip6 was, on the other hand, expressed in both pluripotent ESC stages. These results are in keeping with the qPCR data for the cell lines and the *in vivo* analysis.

We next investigated whether our predictor genes could distinguish between true naïve and primed human PSCs. By comparing the expression levels of these genes in primed-hESCs and their respective reverted-hESCs (Chan et al., 2013; Gafni et al., 2013; Theunissen et al., 2014; Takashima et al., 2014) or in embryo derived naïve-hPSCs (Guo et al., 2016) we found that some of our genes (*SPIC*, *KLF4*, *FST*, *DUSP6*, *LPAR4*, *JAKMIP2* and *CAR14*) readily identified the three lines (Takashima, Theunissen and Guo lines) which have been shown to align better with the pre-implantation monkey embryo (Nakamura et al., 2016). However, not all of our predictor genes were significantly up/downregulated as expected in these lines (Fig. 6C). Thus, we looked at the expression levels of these genes in published data from human pre-implantation embryos (Blakeley et al., 2015) and found that amongst the naïve associate-genes only *GJB5* was not expressed in the pre-implantation epiblast (Fig. S6C), suggesting that either *GJB5* is not conserved in human, or that its expression starts later in the expanded blastocyst but prior to implantation. Of the primed-associated genes, three (*DUSP6*, *FST* and *LPAR4*) are not expressed in human pre-implantation epiblast and two (*CA4* and *JAKMIP2*) are expressed, but at very low levels (Fig. S6C). Interestingly, all of these genes clearly distinguished between naïve (reset and embryo-derived) and primed hESCs (Fig. 6C), confirming these are conserved genes across mammalian species.

Together, these data confirm the genome-wide expression analyses and highlight pluripotent state specific characteristics which could be used as molecular criteria to predict the identity of pluripotent cells *in vitro*.

### Knockdown experiments reveal that some of the naïve/primed ‘predictor’ genes studied affect the maintenance of pluripotency

**Fig. 7.**
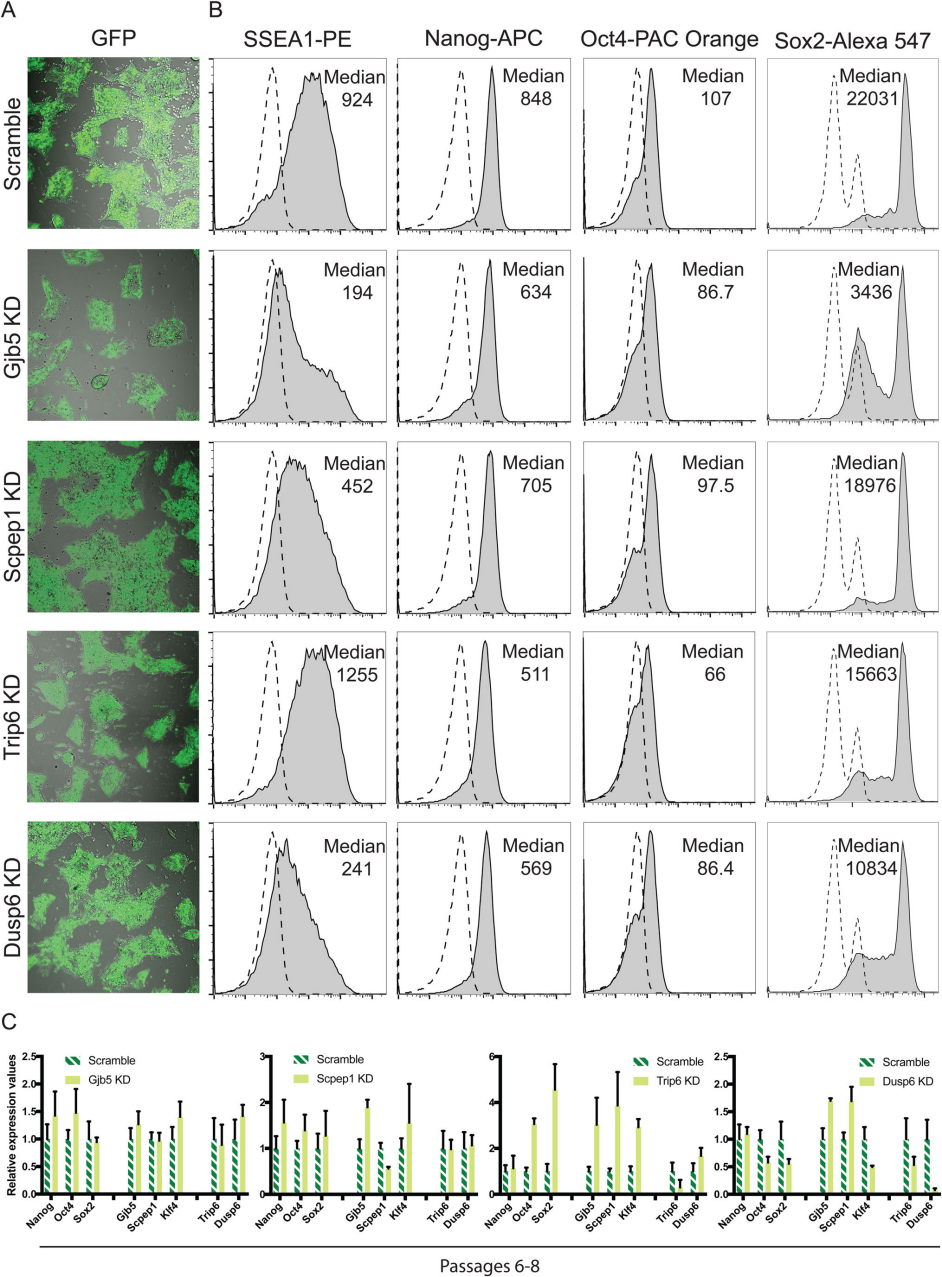
**Pluripotency stability is affected after knocking down some newly identified conserved genes.** (A) Confocal micrographs of mESCs 72 h after transduction showing the levels of GFP+ transduced cells. These are representative results of three biological replicates. (B) Flow cytometry plots showing the levels of pluripotency gene expression in KD mESCs. Median is highlighted for the sample analysed and the respective IgG control is presented in a dashed line. These are representative results of three biological replicates. (C) RT-qPCR showing the expression levels of core pluripotency genes and newly discovered pluripotency-associated genes in knockdown and control scramble transduced mESCs after six to eight passages in 3 μg puromycin. Expression values are normalised against *Gapdh*. Three independent biological replicates were analysed. Error bars represent the standard deviation.

To discover whether the newly identified naïve- or primed-associated genes play a role in controlling pluripotency, we knocked down the expression of *Gjb5*, *Scpep1* and *Dusp6* in 2i-mESCs using a combination of short-hairpin RNAs (Fig. 7). We chose to include Dusp6 and Trip6 in this analysis because these genes are expressed in ground state cells, albeit at lower levels than in primed cells (Fig. 6A). 2i-mESCs were efficiently transduced (Fig. 7A) and gene knockdown was confirmed by PCR analysis 72 h after (Fig. S7A). Knockdown of *Gjb5*, *Scpep1* or *Dusp6*, but not of *Trip6*, led to perturbation of the pluripotent state as demonstrated by the reduced expression of SSEA1 (Fig. 7B). Reduced expression of *Oct4* and *Sox2* was also observed both at the RNA and protein level when the naïve-associated gene *Gjb5* was knocked down (Fig. 7B; Fig. S7A) and we were unable to establish a knockdown cell (KD-cell) line from these cells as they had impaired proliferation capacity (data not shown). In contrast to the other genes tested (Scpep1, Dusp6 and Trip6), the Gjb5 knockdown cells quickly lost the KD-viruses and the associated knockdown of Gjb5 over passaging (Fig. 7C; Fig. S7B), suggesting that the lowly/non-transduced cells take over the culture during prolonged culture. Interestingly, knockdown of the naïve-primed transition-associated gene *Dusp6* also led to the downregulation of *Nanog*, *Oct4* and *Sox2* RNA expression as well as Oct4 and Sox2 protein levels (Fig. 7B; Fig. S7A). Surprisingly, we were able to maintain a Dusp6 KD-cell line and it grossly retained the phenotype observed at 72 h, except for a moderate increase in the expression levels of *Gjb5* and *Scpep1*, confirming its role upstream of *Oct4* and *Sox2* expression (Fig. 7C). The *Scpep1* knockdown did not interfere with the expression of the core pluripotency genes (Fig. 7B; Fig. S7A), suggesting that, unlike *Gjb5* and *Dusp6*, this gene is not involved in the core pluripotency circuitry. We were able to establish a KD-cell line from Scpep1 knockdown cells and these retained the same phenotype observed at 72 h post-transduction (Fig. 7C). However, like the Gjb5 KD-cells, these cells proliferated much slower than wild-type cells or the scramble control cells (data not shown). Lastly, the knockdown of *Trip6* showed reduced protein levels of Nanog, Oct4 and Sox2 which, intriguingly, was not reflected at the RNA level (Fig. 7B; Fig. S7A), suggestive of posttranscriptional modulation of pluripotency factor expression. Trip6 KD-cells were established successfully and proliferated normally. Of note was the fact this line expressed higher levels of *Oct4*, *Sox2*, *Klf4*, *Gjb5* and *Scpep1*, which suggests that despite an initial destabilisation of the pluripotent state (lower levels of Oct4, Sox2 and Nanog at the protein level), this line progressively stabilised in a ‘higher-naïve’ state (Fig. 7C).

Given the phenotype of the Dusp6 and Trip6 KDs in 2i-mESCs, we also determined if these genes impacted on pluripotency in mEpiSCs where they are expressed at higher levels (Fig. S7C). We found that the KD of both *Dusp6* and *Trip6* lead to differentiation as determined by the higher levels of *Sox2*, *Bra* and *Foxa2*. This was accompanied by a very modest decrease of *Nanog* and *Oct4* in the Dusp6 KD, which could reflect the fact we only obtained a low level of KD (40% KD) in these cells and suggests that differentiation in the mEpiSCs Dusp6-KD is likely due to increased levels of FGF/ERK as it is already known that Dusp6 counteracts ERK signalling (Yang et al., 2012).

In conclusion, at least some of the genes discovered through our analysis play a role in maintaining pluripotency. Interestingly, *Dusp6*’s role in pluripotency is not restricted to the pluripotency state it is mainly associated with, but this is not so surprising given that this gene is not exclusively expressed in the primed state. Moreover, in mESCs, the absence of the naïve-prime transition gene *Trip6* appears to promote a ‘higher-naïve’ state of pluripotency, suggesting a link between Trip6 levels of expression and the naïve to primed transition.

## DISCUSSION

Here we compare the transcriptomes of pre-implantation and early post-implantation embryos of bovine, porcine and murine embryos, with the aim of elucidating the molecular basis of pluripotency within these embryos and the extent of conservation of stem cell gene expression patterns between corresponding embryonic stages. We used 1:1:1 orthologues in the analysis to focus on conserved traits and thus identify the cross-species molecular signature for naïve and primed pluripotency. This reductionist approach allowed us to identify with great confidence a small set of molecular determinants for each pluripotent state. We also identified divergences across species. These are likely underestimated because, by focusing on orthologue genes, we ignored genes which have related genes in the different species. Thus, more work needs to be performed to address the inter-species divergences with the aim of explaining differences in stem cell isolation and properties between ungulates and mice.

### ICM to ERSE transition reflects the pluripotent state transition in mammalian development

Our analysis showed that the ICM to ERSE transition is most critical, while the changes taking place between the ERSE and APE stage are either less dramatic or less conserved. Analysis of monkey embryo development highlighted a similar trend where the early to late post-implantation epiblast transition revealed few changes in gene expression (Nakamura et al., 2016). This may underlie the fact that the ICM to ERSE transition is characterised by both the segregation of the primitive endoderm from the epiblast and the conversion from a naïve to a primed pluripotency state (Nichols and Smith, 2009; Brons et al., 2007; Tesar et al., 2007). Conservation of such processes across mammals is strongly indicated by the presence, among the commonly downregulated (ICM-to-ERSE) genes, of some naïve pluripotency genes such as *Tfcp2l1, Klf4* and *Klf5*, and others expressed in the primitive endoderm in the mouse such *as Pdgfra, Gata6* and *Sdc4*. The similarities seen within ERSE and APE stages may also suggest that embryos at these stages are already within the phylotypic stage of embryo development and, as per the hourglass model of embryonic evolution (Varlet et al., 1997), that would indicate that while the ICM stage represents the early most divergent part of the hourglass, the ERSE/APE stage represents the beginning of the ‘narrowing’ part of the hourglass.

### New naïve and primed pluripotency predictor genes were identified

By comparing our *in vivo* data with the expression data for mouse pluripotent stem cell lines, we identified a set of pluripotency predictor genes of which at least some are important for pluripotency maintenance. It was reassuring to find known pluripotency-associated genes, *Klf4* and *Lin28b*, among the differentially expressed gene set. *Klf4* has been previously shown to be activated downstream of the LIF/STAT pathway to induce mESC self-renewal, for restoring naïve pluripotency in EpiSCs (Guo et al., 2009; Hall et al., 2009) and to be required for reprogramming of mouse iPSCs (Wei et al., 2009). *LIN28B* has also been used for reprogramming but, unlike *Klf4*, it was used to reprogram hiPSCs (Yu et al., 2007), which bear more resemblances to mEpiSCs (Brons et al., 2007; Tesar et al., 2007). Furthermore*, Lin28B* has been shown to be a negative regulator of the differentiation-promoting *Let7* miRNAs, which are upregulated in mEpiSCs (Jouneau et al., 2012; Viswanathan et al., 2008). Interestingly, *Lin28b* expression has only been described *in vivo* in pre-implantation mouse embryos (Vogt et al., 2012). Here, we confirmed its expression in murine blastocyst cells but also identified it as being upregulated in murine, bovine and porcine ERSE and APE stage epiblasts.

Other genes highlighted by our analysis have been described as pluripotency-associated genes, but their role in the pluripotency circuitry has not been determined. Amongst those is *Dusp6*, a gene whose expression was enriched at the ERSE/APE stages as well as in serum-mESCs and in mEpiSCs. *Dusp6* belongs to the MAP kinase phosphatase family and was shown to restrain the activity of Erk signalling during the early stages of differentiation (Yang et al., 2012). Furthermore*,* its promoter was shown to contain a pluripotency-specific enhancer (Zhang et al., 2009). Here we further show that when *Dusp6* is knocked down, cells differentiate and the pluripotency circuitry is disrupted since reduced expression of pluripotency genes is observed (Nanog, Oct4 and Sox2). As such, *Dusp6* could be considered a ‘guardian’ of pluripotency. Another example is Trip6, a gene whose expression was enriched in ERSE/APE stages as well as in serum-mESCs and in mEpiSCs. Trip6 was identified previously as being part of the module of genes which are enriched in the preimplantation epiblast and in 2i-mESCs but not in diapause embryos (Boroviak et al., 2014). Here we further demonstrate that Trip6 is a naïve-primed transition gene, which is expressed moderately higher in serum-mESCs and mEpiSCs. Interestingly, when Trip6 is knocked down in 2i-mESCs, these cells appear to transition over passaging into a ‘higher-naïve’ state of pluripotency, as judged by the increased levels of naïve-associated pluripotency genes *Oct4* and *Sox2*.

We also found conserved genes that have never been associated with pluripotency. Amongst these, *Gjb5/GJB5* and *Scpep1/SCPEP1*, which are naïve-associated genes, have been linked with the retinoic acid metabolic pathway, as has the described pluripotency gene *Klf4/KLF4* (Hatakeyama et al., 2011; Chen et al., 2001; Shi et al., 2012). It has been reported that reprogramming of human somatic cells to induced pluripotent stem cells can be achieved more rapidly and efficiently by co-expressing retinoic acid receptor gamma (Wang et al., 2011), which is amongst the genes we found to be upregulated *in vivo* in APE stage embryos and *in vitro* in serum-mESCs and mEpiSCs. The same study also showed that reprogramming with retinoic acid receptor gamma induces naïve-equivalent hiPSCs. As such, it would be of interest to further investigate this pathway in the context of pluripotency maintenance versus differentiation, and generically as a potential pathway for maintaining naïve pluripotency in other species. Here we investigated whether these genes disrupt pluripotency by performing *in vitro* gene knockdowns of *Scpep1* or *Gjb5* and show that the knockdown of both genes (independently) leads to cell differentiation (SSEA1 downregulation) and to reduced proliferation. *Gjb5* in particular appears to be an important gene within the pluripotency circuitry because it is required for normal expression of key pluripotency genes (Oct4 and Sox2), and maintenance of a Gjb5-KD line proved to be difficult. The involvement of Scpep1 in pluripotency is less clear since its knockdown does not interfere with the expression of core pluripotency genes. It is plausible this reflects a role of Scpep1 upstream of *Myc* genes, which are essential for self-renewal maintenance and do not interfere with the expression of core pluripotency genes (Varlakhanova et al., 2010).

The physiological functions of *Sema6a*, *Jakmip2*, and of other ERSE/APE- and mEpiSC-associated genes remain largely unknown. These primed-pluripotency associated genes provide markers that could be used to determine whether cultured ICMs from non-murine species drift towards a late epiblast state. It would therefore be very interesting to track the progression of the human inner cell mass during embryonic stem cell derivation, similar to what was previously done (O'Leary et al., 2012), and assess the expression of the naïve and primed pluripotent-associated genes identified in our study during the process of naïve or conventional primed hESC derivation.

We used our predictor gene set as a molecular criterion to distinguish between human naïve (reset or embryo-derived) and primed pluripotent cells *in vitro* (Chan et al., 2013; Gafni et al., 2013; Theunissen et al., 2014; Takashima et al., 2014; Guo et al., 2016). All the predictor genes were found expressed in both type of cells, but interestingly, only in some of the naïve human PSC lines did our genes follow the up/downregulation trend expected. Of note, some of our genes (*SPIC*, *KLF4*, *FST*, *DUSP6*, *LPAR4*, *JAKMIP2* and *CAR14*) readily identified the three lines (Takashima, Theunissen and Guo lines) which have been shown to align better with the pre-implantation monkey embryo (Nakamura et al., 2016). We searched published human pre-implantation embryo data (Blakeley et al., 2015) for our gene sets and found that all of the primed associated genes (*DUSP6*, *FST*, *LPAR4*, *JAKMIP2* and *CAR14*) which distinguish between naïve and primed hESCs were absent or expressed at very low levels in the human pre-implantation epiblast. Remarkably, we also found that, of our naïve-associated genes, four of them are expressed in the human blastocyst (*SPIC*, *KLF4*, *PSAP* and *SCPEP1*). However, only *SPIC* and *KLF4* are upregulated in naïve hESCs compared to primed hESCs, suggesting that these naïve hESCs have stabilised in an in-between state between the true naïve and the primed state. It has been shown using a range of molecular assays that naïve human PSCs acquire key features of corresponding pluripotent cells *in vivo* but fail to recapitulate the embryonic context entirely (Theunissen et al., 2016). The inappropriate up/downregulation of some of our predictor genes in the three naïve lines which best correlate with the monkey pre-implantation epiblast likely reflect the incomplete reversal of primed pluripotency in these lines (or attainment of naïve pluripotency in the case of the embryo derived naïve hESC line) but we cannot discard the hypothesis that some of these genes are simply not conserved in primates. We propose these (or at least some of these) predictor genes represent a snapshot of the developmental coordinate of pluripotency and could be used as molecular criterion to identify naïve and primed pluripotent cells *in vitro*.

### Developmental stage of mouse PSCs was confirmed

By assessing the correlation of the stem cell lines with the *in vivo* samples as well as looking at the expression gene trends in both *in vivo* and *in vitro* datasets we were able to assess the developmental position of the stem cells. For example, we found genes like *Spic*, of which expression was restricted to ICM and 2i-ESCs. This indicates that, despite the fact that 2i-mESCs do not correlate with ICM stage embryos, they actually share some traits with ICM cells. This was further confirmed by the global correlation and evolutionary conservation analysis of the data. In sum, our findings suggest that the ‘developmental position’ of 2i-mESCs is in between the ICM and ERSE stage epiblast, while serum-ESCs closely resemble the ERSE stage epiblast, which is in keeping with what has been proposed by others (Chen et al., 2016; Boroviak et al., 2014). The ‘developmental position’ of mEpiSCs was less obvious as these cells grouped closely with both pre-gastrulating (ERSE) and early gastrula epiblast cells (APE). However, we observed that there were more genes upregulated in mEpiSCs which were uniquely upregulated in APE stage embryos (rather than those upregulated in ERSE stage embryos) suggesting that, in keeping with Kojima et al., mEpiSCs are developmentally more similar to gastrula-stage embryos (Kojima et al., 2014). This observation differs from what has been proposed by Chen et al. which placed mEpiSCs closer to the E5.5 pre-gastrulation stage embryo (Chen et al., 2016).

### Conclusions and future directions

**Fig. 8.**
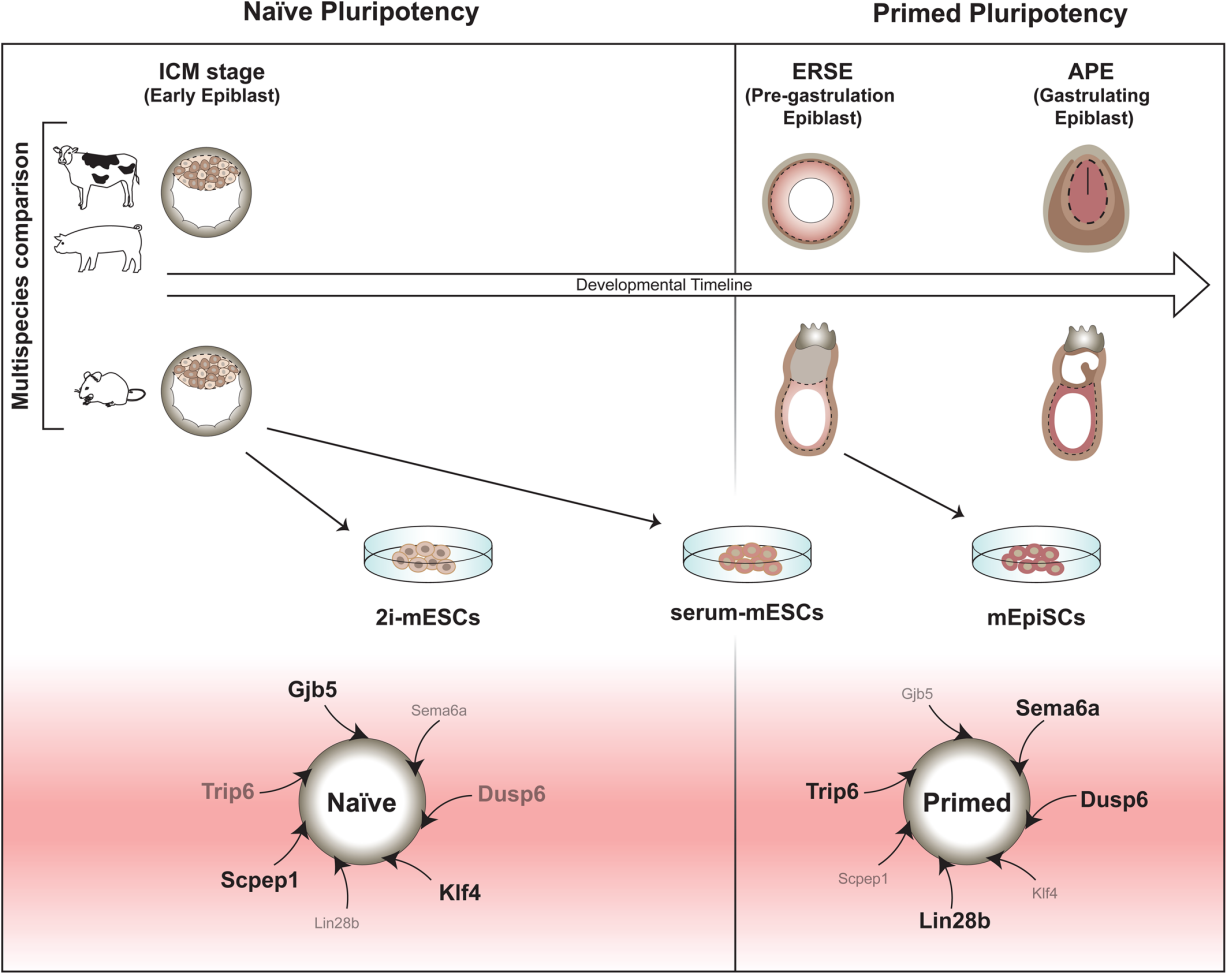
**Proposed model of the developmental timeline of naïve and primed pluripotency in mouse and ungulate species.**

Collectively, the molecular changes we observed in the embryo datasets allowed us to identify: (1) conserved new predictor genes representing a snapshot of the developmental coordinate of pluripotency in mammals (Fig. 8) and which could be used as molecular criteria to identify naïve and primed pluripotent cells *in vitro*; (2) various differences between mouse and ungulate species, regarding the timing of expression of pluripotency markers and signalling effectors and (3) several characteristics of the current ESC systems that are not present in their *in vivo* counterparts. Importantly, we confirmed that while some of our newly identified pluripotency genes play a role in pluripotency *in vitro*, other candidates remain to be studied and could potentially also play important roles in pluripotency. Our dataset could further be used to identify additional cross-species divergences with the aim of trying to circumvent the issues associated with the generation of ESCs from refractory species. Furthermore, understanding the culture adaptations ESC systems undergo *in vitro* is crucial for the efficacy and safety of cell therapies.

## MATERIALS AND METHODS

### Mouse ESC and EpiSC culture

129S2/SvPas and F1 (C57Bl/6JxDBA/2J) mouse ESCs were derived as previously described and cultured using standard protocols (Nagy, 2003). Corresponding 129S2/SvPas and F1 mouse EpiSCs were cultured using standard protocols (Brons et al., 2007). All lines were validated in-house and tested for contaminants. For more details see Supplemental experimental procedures.

### Embryo sample preparation

Both hybrid (B6D2F1) and inbred (129S2) mouse embryos (sex not determined) were used. Pre-implantation mouse samples (ICM stage, E3.5) were collected by flushing the embryos out of the uterus. Post-implantation stage mouse epiblasts were dissected as described (Nagy, 2003) at two time points: at pre-gastrulation stage (ERSE stage, E6.25) and at mid-gastrulation stage (APE stage, E7.25). For details see Supplemental experimental procedures.

Pig embryos (sex not determined) were obtained by breeding Danish Landrace females and Yorkshire males after induced oestrus by weaning. Embryos at E7-E8 (ICM stage), E10-E11 (ERSE stage) and E12-E13 (APE stage) were collected from the uterine horns (for stages E7-E8 and E12-E13) or by manually clipping open the uterine horns for gentle floating off the embryos (E10-E11). For details see Supplemental experimental procedures.

For bovine embryos (sex not determined), ICM stage blastocysts (E7) were obtained after *in vitro* maturation/fertilisation of oocytes and cultured using standard procedures. The ICM was isolated using immunosurgery. To obtain later stages, donor cows were bred by artificial insemination after induced oestrus and super-ovulation. Embryos at E14 (ERSE stage) and at E17 (APE stage) were collected non-surgically by gentle flushing. For details see Supplemental experimental procedures.

### mRNA preparation, cDNA synthesis and sequencing

Pure epiblasts or ICM were harvested into TriZol and snap frozen. RNA was isolated using the PureLink™ RNA Micro Kit (Invitrogen). cDNA was prepared for RTqPCR using superscript III (Invitrogen); or for sequencing using either 100 pg of RNA and following the SMARTer (Illumina, San Diego, USA), Spia-Ovation (NuGEN, Leek, The Netherlands) or oligo dT [adapted from (Kurimoto et al., 2006)] method, or using 1 μg RNA and following the standard random hexamer method from Illumina. The samples were sequenced using an Illumina HiSeq2000. All RNA-seq (FASTQ and BedGraph files) are present in the NCBI GEO SuperSeries GSE53387. For details, see Table S1 and Supplemental experimental procedures.

### Bioinformatic analysis

Details of the bioinformatic analysis performed are described in the Supplemental experimental procedures.

### Real time PCR

Quantitative polymerase chain reactions (q-PCR) were performed in triplicates using SYBR Green ReadyMix (Roche Products Limited, Welwyn Garden City, UK or Applied Biosytems, Foster City, USA) and the results were normalised to the house keeping gene *Gapdh*/*GAPDH* as internal reference (primers supplied by Sigma-Aldrich). For more details, see Supplemental experimental procedures.

### *In situ* hybridisation

Embryos were fixed in 4% paraformaldehyde (PFA; Sigma-Aldrich), dehydrated and stored in methanol 100% at −20°C until further processing. For whole-mount ISH, samples were rehydrated, and processed using a standard protocol. For details, see Supplemental experimental procedures.

### Immunohistochemistry

Mouse embryos, mESCs and mEpiSCs were fixed in 4% (w/v) PFA and immunostained using a standard protocol. For details on antibodies see Supplemental experimental procedures.

### Transduction experiments

2i-mESCs were transduced with sh-RNAs expressing lentiviruses. A control GFP expressing lentivirus was used to test for transduction efficiency. Cells were harvested 72 h after transduction.

### Flow cytometry

Cell suspensions were fixed and stained using the Cytofix-Cytoperm kit (BD Biosciences) and following manufacturer's instructions as described in Supplemental experimental procedures. Cells were analysed using a Beckman Coulter CyAn_ADP_ flow cytometer and FlowJo software (Becton Dickinson, Franklin Lake, USA).

### Accession numbers

All RNA-seq (FASTQ and BedGraph files) are present in the NCBI GEO SuperSeries GSE53387.

### Other datasets used

RNA-seq data for three ESC lines cultured in 2i+Lif and three ESC lines cultured in serum+Lif were obtained from Marks et al. (2012). RNA-seq data for three EpiSCs lines were from Veillard et al. (2014).

## Supplementary Material

Supplementary information

## References

[BIO033282C1] Alberio R., Croxall N. and Allegrucci C. (2010). Pig epiblast stem cells depend on activin/nodal signaling for pluripotency and self-renewal. *Stem Cells Dev.* 19, 1627-1636. 10.1089/scd.2010.0012 20210627 PMC3129689

[BIO033282C2] Artus J., Panthier J. J. and Hadjantonakis A. K. (2010). A role for PDGF signaling in expansion of the extra-embryonic endoderm lineage of the mouse blastocyst. *Development* 137, 3361-3372. 10.1242/dev.050864 20826533 PMC2947752

[BIO033282C3] Avilion A. A., Nicolis S. K., Pevny L. H., Perez L., Vivian N. and Lovell-Badge R. (2003). Multipotent cell lineages in early mouse development depend on SOX2 function. *Genes Dev.* 17, 126-140. 10.1101/gad.224503 12514105 PMC195970

[BIO033282C4] Blakeley P., Fogarty N. M. E., Del Valle I., Wamaitha S. E., Hu T. X., Elder K., Snell P., Christie L., Robson P. and Niakan K. K. (2015). Defining the three cell lineages of the human blastocyst by single-cell RNA-seq. *Development* 142, 3151-3165. 10.1242/dev.123547 26293300 PMC4582176

[BIO033282C5] Boroviak T., Loos R., Bertone P., Smith A. and Nichols J. (2014). The ability of inner-cell-mass cells to self-renew as embryonic stem cells is acquired following epiblast specification. *Nat. Cell Biol.* 16, 513-525. 10.1038/ncb2965 PMC4878656 24859004

[BIO033282C6] Bourillot P. Y., Aksoy I., Schreiber V., Wianny F., Schulz H., Hummel O., Hubner N. and Savatier P. (2009). Novel STAT3 target genes exert distinct roles in the inhibition of mesoderm and endoderm differentiation in cooperation with Nanog. *Stem Cells* 27, 1760-1771. 10.1002/stem.110 19544440

[BIO033282C7] Brons I. G., Smithers L. E., Trotter M. W. B., Rugg-Gunn P., Sun B., Chuva De Sousa Lopes S. M., Howlett S. K., Clarkson A., Ahrlund-Richter L., Pedersen R. A. et al. (2007). Derivation of pluripotent epiblast stem cells from mammalian embryos. *Nature* 448, 191-195. 10.1038/nature05950 17597762

[BIO033282C8] Chambers I., Colby D., Robertson M., Nichols J., Lee S., Tweedie S. and Smith A. (2003). Functional expression cloning of Nanog, a pluripotency sustaining factor in embryonic stem cells. *Cell* 113, 643-655. 10.1016/S0092-8674(03)00392-1 12787505

[BIO033282C9] Chan Y.-S., Göke J., Ng J.-H., Lu X., Gonzales K. A. U., Tan C.-P., Tng W.-Q., Hong Z.-Z., Lim Y.-S. and Ng H.-H. (2013). Induction of a human pluripotent state with distinct regulatory circuitry that resembles preimplantation epiblast. *Cell Stem Cell* 13, 663-675. 10.1016/j.stem.2013.11.015 24315441

[BIO033282C10] Chazaud C., Yamanaka Y., Pawson T. and Rossant J. (2006). Early lineage segregation between epiblast and primitive endoderm in mouse blastocysts through the Grb2-MAPK pathway. *Dev. Cell* 10, 615-624. 10.1016/j.devcel.2006.02.020 16678776

[BIO033282C11] Chen J., Streb J. W., Maltby K. M., Kitchen C. M. and Miano J. M. (2001). Cloning of a novel retinoid-inducible serine carboxypeptidase from vascular smooth muscle cells. *J. Biol. Chem.* 276, 34175-34181. 10.1074/jbc.M104162200 11447226

[BIO033282C12] Chen G., Schell J. P., Benitez J. A., Petropoulos S., Yilmaz M., Reinius B., Alekseenko Z., Shi L., Hedlund E., Lanner F. et al. (2016). Single-cell analyses of X Chromosome inactivation dynamics and pluripotency during differentiation. *Genome Res.* 26, 1342-1354. 10.1101/gr.201954.115 27486082 PMC5052059

[BIO033282C13] Cockburn K. and Rossant J. (2010). Making the blastocyst: lessons from the mouse. *J. Clin. Invest.* 120, 995-1003. 10.1172/JCI41229 20364097 PMC2846056

[BIO033282C14] Darr H. and Benvenisty N. (2009). Genetic analysis of the role of the reprogramming gene LIN-28 in human embryonic stem cells. *Stem Cells* 27, 352-362. 10.1634/stemcells.2008-0720 19038789

[BIO033282C15] Degrelle S. A., Campion E., Cabau C., Piumi F., Reinaud P., Richard C., Renard J.-P. and Hue I. (2005). Molecular evidence for a critical period in mural trophoblast development in bovine blastocysts. *Dev. Biol.* 288, 448-460. 10.1016/j.ydbio.2005.09.043 16289134

[BIO033282C16] Dowell K. G., Simons A. K., Bai H., Kell B., Wang Z. Z., Yun K. and Hibbs M. A. (2014). Novel insights into embryonic stem cell self-renewal revealed through comparative human and mouse systems biology networks. *Stem Cells* 32, 1161-1172. 10.1002/stem.1612 24307629 PMC4404315

[BIO033282C17] Gafni O., Weinberger L., Mansour A. A. F., Manor Y. S., Chomsky E., Ben-Yosef D., Kalma Y., Viukov S., Maza I., Zviran A. et al. (2013). Derivation of novel human ground state naive pluripotent stem cells. *Nature* 504, 282-286. 10.1038/nature12745 24172903

[BIO033282C18] Guo G., Yang J., Nichols J., Hall J. S., Eyres I., Mansfield W. and Smith A. (2009). Klf4 reverts developmentally programmed restriction of ground state pluripotency. *Development* 136, 1063-1069. 10.1242/dev.030957 19224983 PMC2685927

[BIO033282C19] Guo G., Von Meyenn F., Santos F., Chen Y., Reik W., Bertone P., Smith A. and Nichols J. (2016). Naive pluripotent stem cells derived directly from isolated cells of the human inner cell mass. *Stem Cell Reports* 6, 437-446. 10.1016/j.stemcr.2016.02.005 26947977 PMC4834040

[BIO033282C20] Hall J., Guo G., Wray J., Eyres I., Nichols J., Grotewold L., Morfopoulou S., Humphreys P., Mansfield W., Walker R. et al. (2009). Oct4 and LIF/Stat3 additively induce Kruppel factors to sustain embryonic stem cell self-renewal. *Cell Stem Cell* 5, 597-609. 10.1016/j.stem.2009.11.003 19951688

[BIO033282C21] Hatakeyama S., Mikami T., Habano W. and Takeda Y. (2011). Expression of connexins and the effect of retinoic acid in oral keratinocytes. *J. Oral Sci.* 53, 327-332. 10.2334/josnusd.53.327 21959660

[BIO033282C22] Huang Y., Osorno R., Tsakiridis A. and Wilson V. (2012). In Vivo differentiation potential of epiblast stem cells revealed by chimeric embryo formation. *Cell Rep.* 2, 1571-1578. 10.1016/j.celrep.2012.10.022 23200857

[BIO033282C23] Jouneau A., Ciaudo C., Sismeiro O., Brochard V., Jouneau L., Vandormael-Pournin S., Coppee J.-Y., Zhou Q., Heard E., Antoniewski C. et al. (2012). Naive and primed murine pluripotent stem cells have distinct miRNA expression profiles. *RNA* 18, 253-264. 10.1261/rna.028878.111 22201644 PMC3264912

[BIO033282C24] Kageyama S., Liu H., Nagata M. and Aoki F. (2006). The role of ETS transcription factors in transcription and development of mouse preimplantation embryos. *Biochem. Biophys. Res. Commun.* 344, 675-679. 10.1016/j.bbrc.2006.03.192 16630543

[BIO033282C25] Kojima Y., Kaufman-Francis K., Studdert J. B., Steiner K. A., Power M. D., Loebel D. A. F., Jones V., Hor A., de Alencastro G., Logan G. J. et al. (2014). The transcriptional and functional properties of mouse epiblast stem cells resemble the anterior primitive streak. *Cell Stem Cell* 14, 107-120. 10.1016/j.stem.2013.09.014 24139757

[BIO033282C26] Kurimoto K., Yabuta Y., Ohinata Y., Ono Y., Uno K. D., Yamada R. G., Ueda H. R. and Saitou M. (2006). An improved single-cell cDNA amplification method for efficient high-density oligonucleotide microarray analysis. *Nucleic Acids Res.* 34, e42 10.1093/nar/gkl050 16547197 PMC1409679

[BIO033282C27] Lawson K. A., Meneses J. J. and Pedersen R. A. (1991). Clonal analysis of epiblast fate during germ layer formation in the mouse embryo. *Development* 113, 891-911. 1821858 10.1242/dev.113.3.891

[BIO033282C28] Maddox-Hyttel P., Alexopoulos N., Vajta G., Lewis I., Rogers P., Cann L., Callesen H., Tveden-Nyborg P. and Trounson A. (2003). Immunohistochemical and ultrastructural characterization of the initial post-hatching development of bovine embryos. *Reproduction* 125, 607-623. 10.1530/rep.0.1250607 12683931

[BIO033282C29] Marks H., Kalkan T., Menafra R., Denissov S., Jones K., Hofemeister H., Nichols J., Kranz A., Stewart A. F., Smith A. et al. (2012). The transcriptional and epigenomic foundations of ground state pluripotency. *Cell* 149, 590-604. 10.1016/j.cell.2012.03.026 22541430 PMC3398752

[BIO033282C30] Martello G., Sugimoto T., Diamanti E., Joshi A., Hannah R., Ohtsuka S., Göttgens B., Niwa H. and Smith A. (2012). Esrrb is a pivotal target of the Gsk3/Tcf3 axis regulating embryonic stem cell self-renewal. *Cell Stem Cell* 11, 491-504. 10.1016/j.stem.2012.06.008 23040478 PMC3465555

[BIO033282C31] Masaki H., Kato-Itoh M., Takahashi Y., Umino A., Sato H., Ito K., Yanagida A., Nishimura T., Yamaguchi T., Hirabayashi M. et al. (2016). Inhibition of apoptosis overcomes stage-related compatibility barriers to chimera formation in mouse embryos. *Cell Stem Cell* 19, 587-592. 10.1016/j.stem.2016.10.013 27814480

[BIO033282C32] Nagy A. (2003). *Manipulating the Mouse Embryo: a Laboratory Manual*, Vol. 764, 3rd edn Cold Spring Harbor, N.Y: Cold Spring Harbor Laboratory Press.

[BIO033282C33] Nakamura T., Okamoto I., Sasaki K., Yabuta Y., Iwatani C., Tsuchiya H., Seita Y., Nakamura S., Yamamoto T. and Saitou M. (2016). A developmental coordinate of pluripotency among mice, monkeys and humans. *Nature* 537, 57-62. 10.1038/nature19096 27556940

[BIO033282C34] Ng H.-H. and Surani M. A. (2011). The transcriptional and signalling networks of pluripotency. 13, 490-496. 10.1038/ncb0511-490 21540844

[BIO033282C35] Nichols J. and Smith A. (2009). Naive and primed pluripotent states. *Cell Stem Cell* 4, 487-492. 10.1016/j.stem.2009.05.015 19497275

[BIO033282C36] O'Leary T., Heindryckx B., Lierman S., Van Bruggen D., Goeman J. J., Vandewoestyne M., Deforce D., De Sousa Lopes S. M. C. and De Sutter P. (2012). Tracking the progression of the human inner cell mass during embryonic stem cell derivation. *Nat. Biotechnol.* 30, 278-282. 10.1038/nbt.2135 22371082

[BIO033282C37] Osorno R. and Chambers I. (2011). Transcription factor heterogeneity and epiblast pluripotency. *Philos. Trans. R. Soc. Lond. B Biol. Sci.* 366, 2230-2237. 10.1098/rstb.2011.0043 21727128 PMC3130424

[BIO033282C38] Pauklin S., Pedersen R. A. and Vallier L. (2011). Mouse pluripotent stem cells at a glance. *J. Cell Sci.* 124, 3727-3732. 10.1242/jcs.074120 22124139

[BIO033282C39] Pelton T. A., Sharma S., Schulz T. C., Rathjen J. and Rathjen P. D. (2002). Transient pluripotent cell populations during primitive ectoderm formation: correlation of in vivo and in vitro pluripotent cell development. *J. Cell Sci.* 115, 329-339. 11839785 10.1242/jcs.115.2.329

[BIO033282C40] Qi X., Li T.-G., Hao J., Hu J., Wang J., Simmons H., Miura S., Mishina Y. and Zhao G.-Q. (2004). BMP4 supports self-renewal of embryonic stem cells by inhibiting mitogen-activated protein kinase pathways. *Proc. Natl. Acad. Sci. USA* 101, 6027-6032. 10.1073/pnas.0401367101 15075392 PMC395917

[BIO033282C41] Renfree M. B. and Fenelon J. C. (2017). The enigma of embryonic diapause. *Development* 144, 3199-3210. 10.1242/dev.148213 28928280

[BIO033282C42] Russell R., Ilg M., Lin Q., Wu G., Lechel A., Bergmann W., Eiseler T., Linta L., Kumar P. P., Klingenstein M. et al. (2015). A dynamic role of tbx3 in the pluripotency circuitry. *Stem Cell Reports* 5, 1155-1170. 10.1016/j.stemcr.2015.11.003 26651606 PMC4682344

[BIO033282C43] Shi J. H., Zheng B., Chen S., Ma G. Y. and Wen J. K. (2012). Retinoic acid receptor alpha mediates all-trans-retinoic acid-induced Klf4 gene expression by regulating Klf4 promoter activity in vascular smooth muscle cells. *J. Biol. Chem.* 287, 10799-10811. 10.1074/jbc.M111.321836 22337869 PMC3322846

[BIO033282C144] Takahashi K., Mitsui K. and Yamanaka S. (2003). Role of ERas in promoting tumour-like properties in mouse embryonic stem cells. *Nature* **423**, 541-545 10.1038/nature01646 12774123

[BIO033282C44] Takashima Y., Guo G., Loos R., Nichols J., Ficz G., Krueger F., Oxley D., Santos F., Clarke J., Mansfield W. et al. (2014). Resetting transcription factor control circuitry toward ground-state pluripotency in human. *Cell* 158, 1254-1269. 10.1016/j.cell.2014.08.029 25215486 PMC4162745

[BIO033282C45] Tesar P. J., Chenoweth J. G., Brook F. A., Davies T. J., Evans E. P., Mack D. L., Gardner R. L. and Mckay R. D. G. (2007). New cell lines from mouse epiblast share defining features with human embryonic stem cells. *Nature* 448, 196-199. 10.1038/nature05972 17597760

[BIO033282C46] Theunissen T. W., Powell B. E., Wang H., Mitalipova M., Faddah D. A., Reddy J., Fan Z. P., Maetzel D., Ganz K., Shi L. et al. (2014). Systematic identification of culture conditions for induction and maintenance of naive human pluripotency. *Cell Stem Cell* 15, 471-487. 10.1016/j.stem.2014.07.002 25090446 PMC4184977

[BIO033282C47] Theunissen T. W., Friedli M., He Y., Planet E., O'Neil R. C., Markoulaki S., Pontis J., Wang H., Iouranova A., Imbeault M. et al. (2016). Molecular Criteria for Defining the Naive Human Pluripotent State. *Cell Stem Cell* 19, 502-515. 10.1016/j.stem.2016.06.011 27424783 PMC5065525

[BIO033282C48] Van Leeuwen J., Berg D. K., Smith C. S., Wells D. N. and Pfeffer P. L. (2014). Specific epiblast loss and hypoblast impairment in cattle embryos sensitized to survival signalling by ubiquitous overexpression of the proapoptotic gene BAD. *PLoS ONE* 9, e96843 10.1371/journal.pone.0096843 24806443 PMC4013130

[BIO033282C49] Varlakhanova N. V., Cotterman R. F., Devries W. N., Morgan J., Donahue L. R., Murray S., Knowles B. B. and Knoepfler P. S. (2010). myc maintains embryonic stem cell pluripotency and self-renewal. *Differentiation* 80, 9-19. 10.1016/j.diff.2010.05.001 20537458 PMC2916696

[BIO033282C50] Varlet I., Collignon J. and Robertson E. J. (1997). nodal expression in the primitive endoderm is required for specification of the anterior axis during mouse gastrulation. *Development* 124, 1033-1044. 9056778 10.1242/dev.124.5.1033

[BIO033282C51] Veillard A. C., Marks H., Bernardo A. S., Jouneau L., Laloë D., Boulanger L., Kaan A., Brochard V., Tosolini M., Pedersen R. et al. (2014). Stable methylation at promoters distinguishes epiblast stem cells from embryonic stem cells and the in vivo epiblasts. *Stem Cells Dev.* 23, 2014-2029. 10.1089/scd.2013.0639 24738887 PMC4142781

[BIO033282C52] Viswanathan S. R., Daley G. Q. and Gregory R. I. (2008). Selective blockade of microRNA processing by Lin28. *Science* 320, 97-100. 10.1126/science.1154040 18292307 PMC3368499

[BIO033282C53] Vogt E. J., Meglicki M., Hartung K. I., Borsuk E. and Behr R. (2012). Importance of the pluripotency factor LIN28 in the mammalian nucleolus during early embryonic development. *Development* 139, 4514-4523. 10.1242/dev.083279 23172912 PMC3912245

[BIO033282C54] Wang W., Yang J., Liu H., Lu D., Chen X., Zenonos Z., Campos L. S., Rad R., Guo G., Zhang S. et al. (2011). Rapid and efficient reprogramming of somatic cells to induced pluripotent stem cells by retinoic acid receptor gamma and liver receptor homolog 1. *Proc. Natl. Acad. Sci. USA* 108, 18283-18288. 10.1073/pnas.1100893108 21990348 PMC3215025

[BIO033282C55] Wei Z., Yang Y., Zhang P., Andrianakos R., Hasegawa K., Lyu J., Chen X., Bai G., Liu C., Pera M. et al. (2009). Klf4 interacts directly with Oct4 and Sox2 to promote reprogramming. *Stem Cells* 27, 2969-2978. 10.1002/stem.231 19816951

[BIO033282C56] Yang S. H., Kalkan T., Morrisroe C., Smith A. and Sharrocks A. D. (2012). A genome-wide RNAi screen reveals MAP kinase phosphatases as key ERK pathway regulators during embryonic stem cell differentiation. *PLoS Genet.* 8, e1003112 10.1371/journal.pgen.1003112 23271975 PMC3521700

[BIO033282C157] Ye S., Li P., Tong C. and Ying Q.-L. (2013). Embryonic stem cell self-renewal pathways converge on the transcription factor Tfcp2l1. *The EMBO journal* **32**, 2548-2560 10.1038/emboj.2013.175 PMC3791365 23942238

[BIO033282C57] Ying Q.-L., Wray J., Nichols J., Batlle-Morera L., Doble B., Woodgett J., Cohen P. and Smith A. (2008). The ground state of embryonic stem cell self-renewal. *Nature* 453, 519-523. 10.1038/nature06968 18497825 PMC5328678

[BIO033282C58] Yu J., Vodyanik M. A., Smuga-Otto K., Antosiewicz-Bourget J., Frane J. L., Tian S., Nie J., Jonsdottir G. A., Ruotti V., Stewart R. et al. (2007). Induced pluripotent stem cell lines derived from human somatic cells. *Science* 318, 1917-1920. 10.1126/science.1151526 18029452

[BIO033282C59] Zhang J. nomura J., Maruyama M., Nishimoto M., Muramatsu M. and Okuda A. (2009). Identification of an ES cell pluripotent state-specific DUSP6 enhancer. *Biochem. Biophys. Res. Commun.* 378, 319-323. 10.1016/j.bbrc.2008.11.068 19032937

