# Supplementary material for: Mammalian embryo comparison identifies novel pluripotency genes associated with the naïve or primed state
Source: Biol Open. 2018 Jul 19;7(8):bio033282. doi: 10.1242/bio.033282 (PMC6124576; doi:10.1242/bio.033282)
Supplement: Supplementary information [file biolopen-7-033282-s1.pdf]

Supplementary Material

I. Supplemental Figures

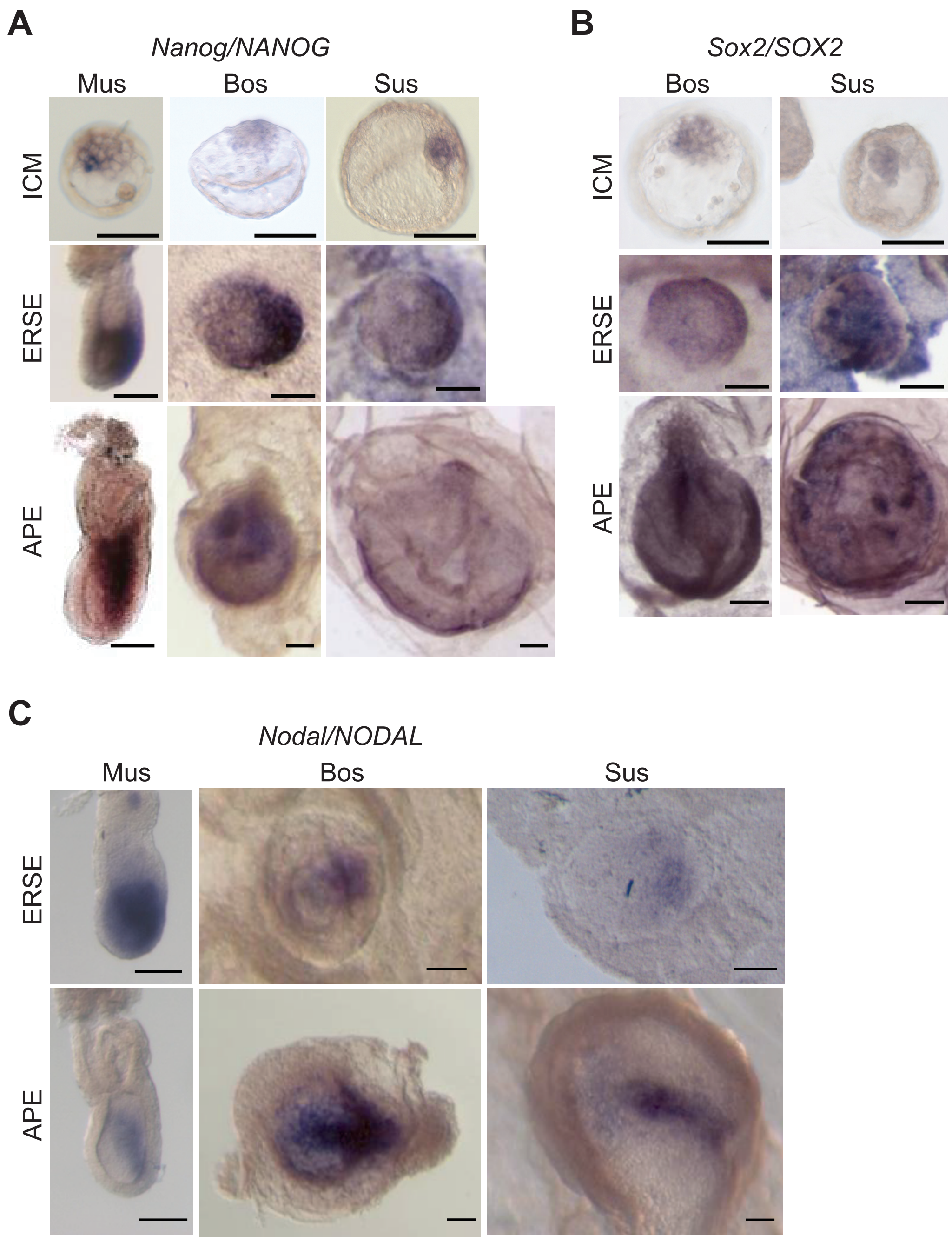

Supplementary Figure 1 related to Figure 1: Whole mount In situ hybridization for **A** Nanog/NANOG, **B** SOX2 and **C** Nodal/NODAL. Scale bar: 100 µm. NANOG and SOX2 staining was weak in the Bos and Sus ICM and not detectable in all of the processed blastocysts. 2-6 embryos were analysed per species and these are representative embryos.

A

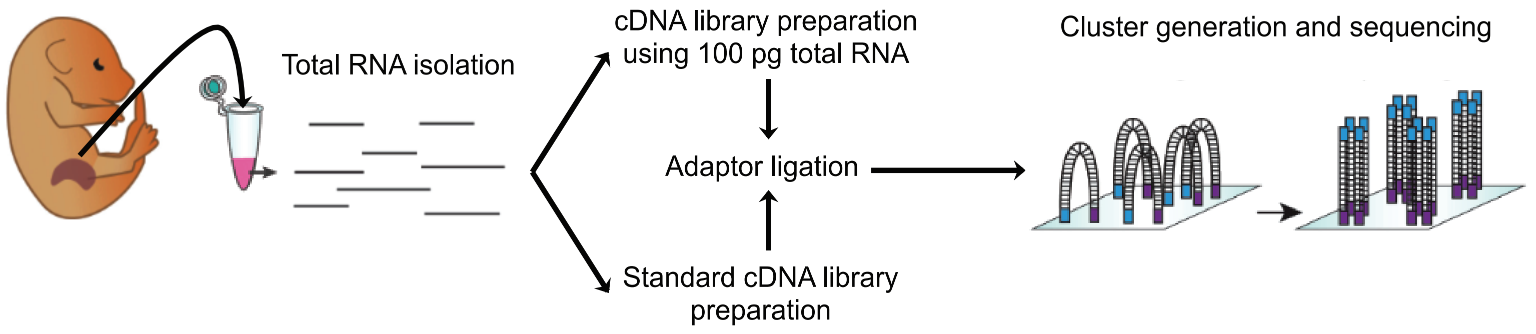

B

Dataset	Reads No.	Mapped No.		rRNA No.		Exonic No.	
Standard - hex	17292980	10237982	59,2%	244845	1,4%	7801089	45,1%
Bernardo/Kurimoto - oligo(dT)	31625942	13563433	42,9%	30009	0,1%	12537781	39,6%
NuGen Ovation - SPIA	29692869	18710146	63,0%	977480	3,3%	7707869	26,0%
Clontech SMARTer - oligo(dT)	30775197	19824071	64,4%	3682058	12,0%	14473753	47,0%

C D E F

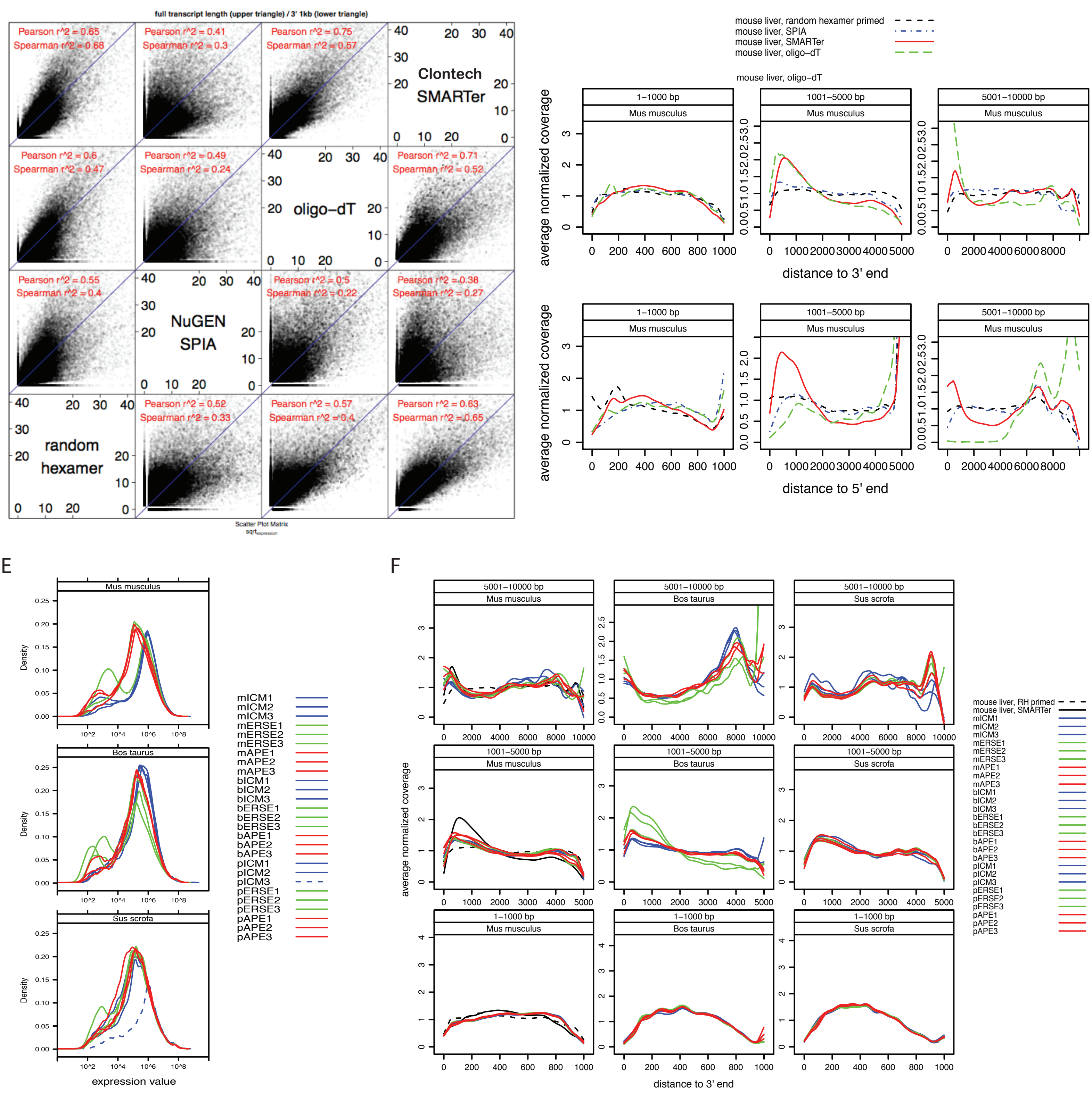

Biology Open • Supplementary information

Supplementary Figure 2 related to Figure 2: A mouse embryonic liver sample was used as a benchmark for testing different methods of RNA amplification. **A** Diagram showing how the comparison between amplification protocols and the standard random hexamer protocol was performed using a liver sample. RNA from this sample was either processed for sequencing using a "macro" method, or 100 pg of total RNA was amplified first using a "micro"-method and subsequently sequenced. **B** Table showing the total amount of reads for the liver sample obtained upon sequencing per protocol used and their respective mappable reads, including those that map to exonic regions or which correspond to ribosomal RNA. **C** Scatter plot matrix of the square-root transformed and normalized expression values of the diluted liver samples. The SMARTer protocol produced the results with the highest correlation to the standard random-hexamer primed protocol of Illumina on undiluted data. The upper-left part of the matrix shows the expression values derived from 1 kb upstream the 3' end of the transcripts, the lower-right part shows the expression derived from full length transcripts. **D** The sidewise coverage plots show reads across the full length of spliced transcripts of 1-5 kb length with a slight bias towards the 3' end that is expected for an oligo-dT priming based method like SMARTer. For these figures all transcripts were aligned by their 3' ends (top) or 5' ends (bottom). The coverage values were calculated according to the formula from the full length coverage of genes by reads. That is, for each position (i = distance to end) the square-root of the number of reads covering the position (ni) was normalized by the number of transcripts covering the position (ti) and squared. The value was then normalized by the total number of bases covering the region (N) in millions to account for the differences in read numbers per sample. Shown are smoothed curves (R function loess() with span=1/10, degree=1, evaluations=2000). **E** Densityplots of the normalized embryonic samples. Note that sample pICM3 has a very low number of mappable reads and was excluded from further analyses. **F** Coverage per site for the final embryonic samples. All transcripts were aligned by their 3' ends as described in Sup. Fig. 2 D.

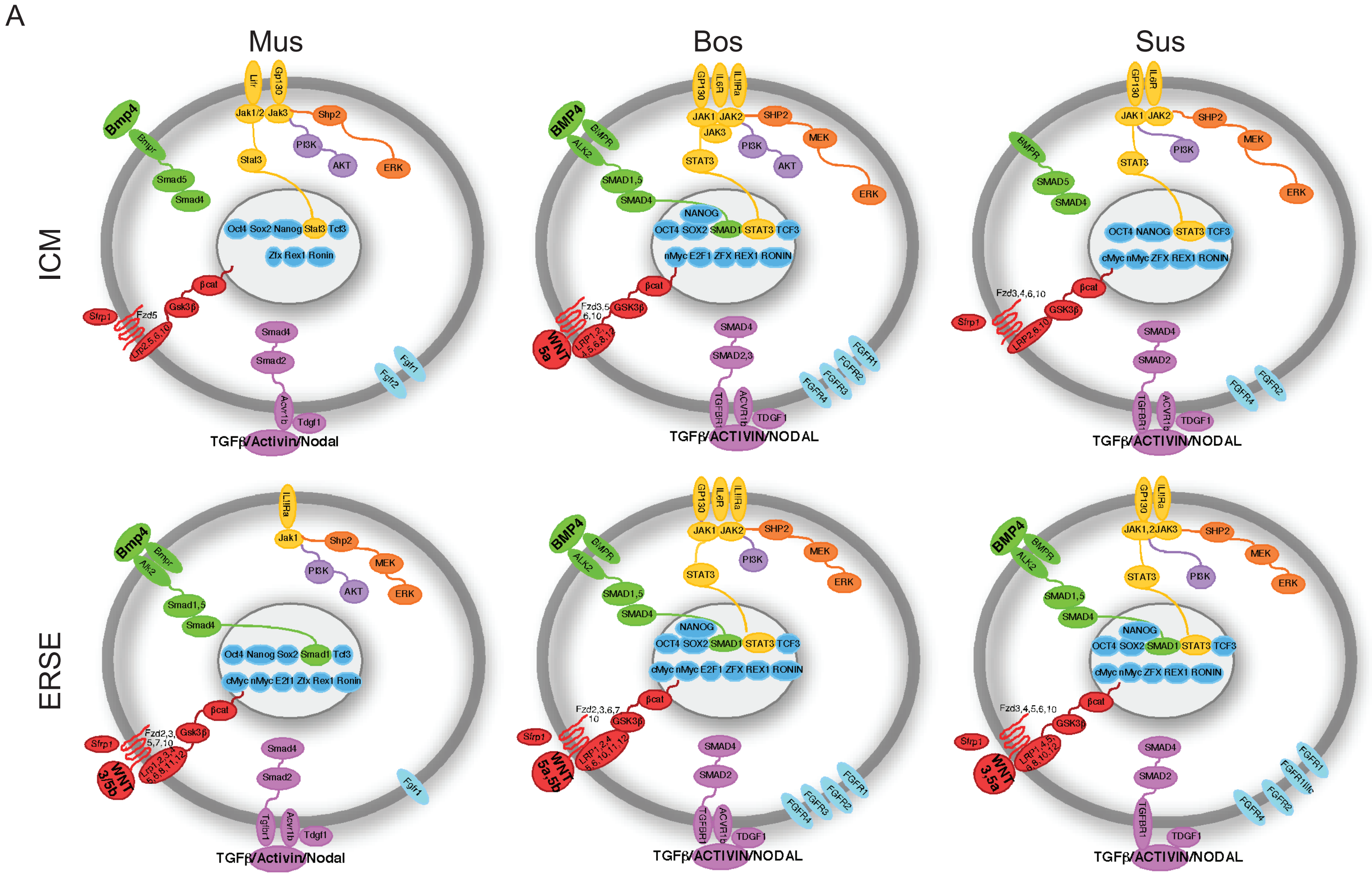

Supplementary Figure 3 related to Figure 3: A Schematic diagram showing the components of known pluripotency active pathways and key pluripotency transcription factors which are expressed (in at least 2/3 samples) in the ICM and ERSE stages in our datasets.

Biology Open • Supplementary information

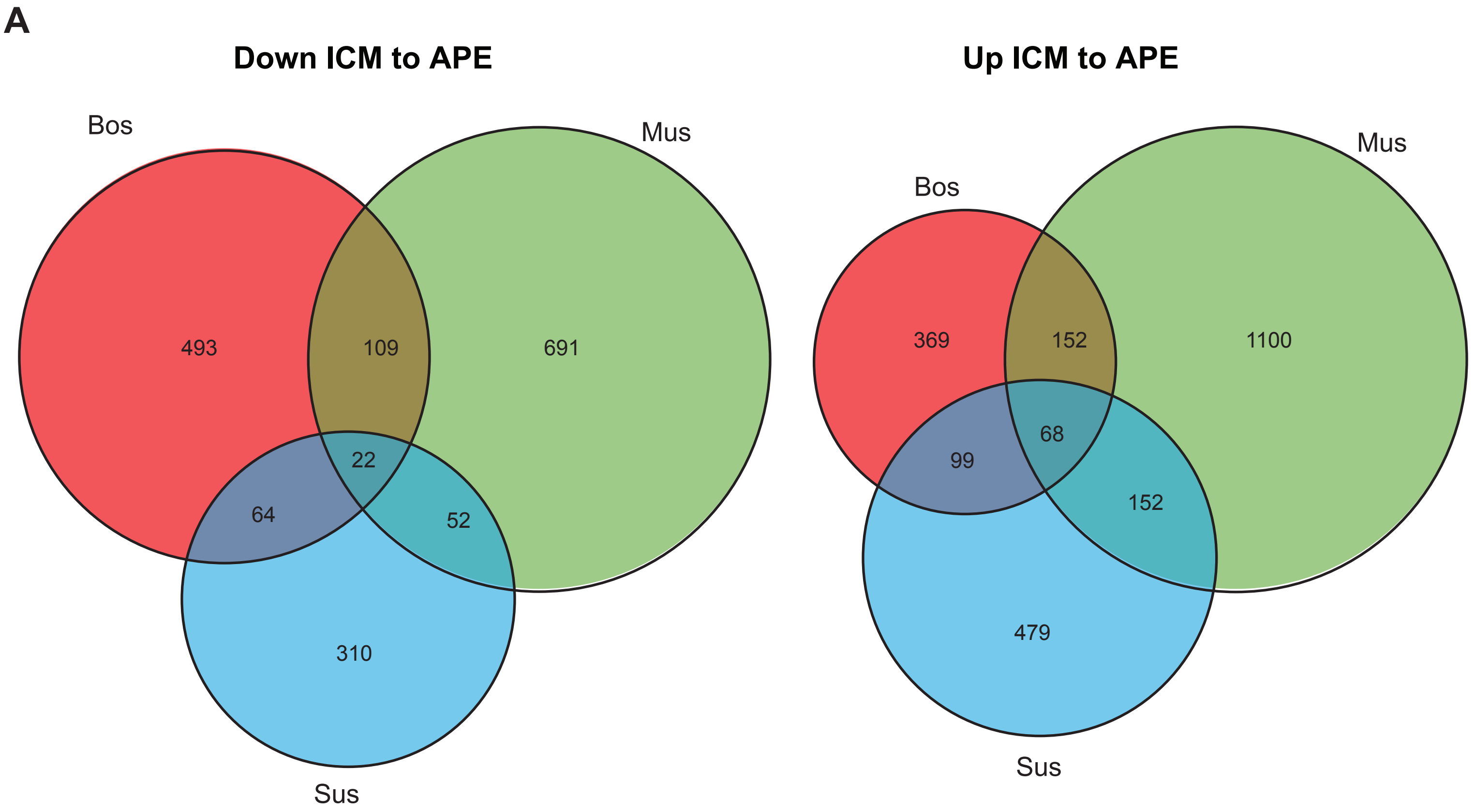

Up ICM to APE	2900011O08Rik; 3632451O06Rik; 4930455F23Rik; Acsl4; Adh5; Arhgef2; Arl3; BC048355; Car14; Cdca7l; Cnn1; Crabp2; Crygb; Dbc1; Dctpp1; Ddt; Ddx26b; Dlg2; Dnmt3b; Dusp6; Eras; Fst; G3bp2; Gabra4; Gatm; Gstm5; Gstt1; Hells; Jakmip2; Jmjd1c; Kcnk1; Kcnt2; Krtcap3; Lin28b; Lmo3; Lmo7; Lpar4; Maged1; Maoa; Mfap2; Myl7; Myo10; Nefm; Nell2; Ngfrap1; Nnat; Phc1; Pls3; Psmb4; Psmb6; Psmd8; Qser1; Rarg; Rbp1; Rfc3; Sdc2; Sept6; Serpini1; Slc38a1; Slc38a5; Smarca1; Ssbp2; Tmem59l; Trip6; Tspan3; Usp44; Zdhhc15; Zfp664
Down ICM to APE	Aqp3; Bcas3; Bcl2l14; Ctsa; Enpep; Fn1; Gjb5; Gm2a; Gpcpd1; Kif13b; Klf4; Klf5; Lcp1; Pdxk; Psap; Rarb; Rnd3; Scpep1; Sdc4; Spic; Stxbp2; Tagln2

Supplementary Figure 4 related to Figure 4: Venn diagrams showing differential expression between ICM and APE stage samples in the three species (with q value ≤ 0.25, minimum fold change 1.5). The genes belonging to the central territory of the diagram are shown below each Venn Diagram.

Biology Open • Supplementary information

Biology Open (2018): doi:10.1242/bio.033282: Supplementary information

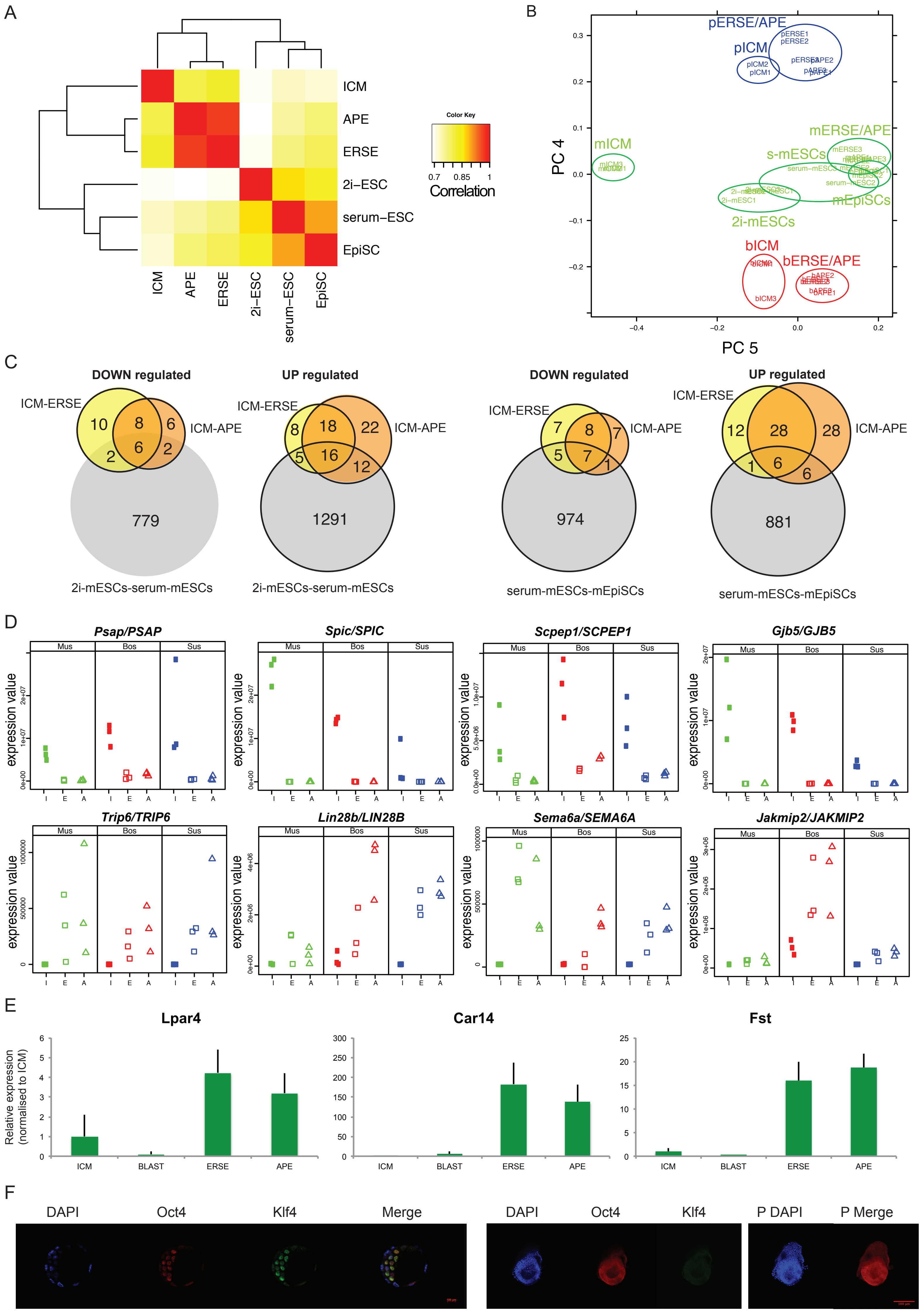

Biology Open • Supplementary information

Supplementary Figure 5 related to Figure 5: A Heat map of the correlations between all in vivo and in vitro samples with their average linkage clustering. **B** Projection of the samples on the principle components 4 and 5. **C** Venn diagrams showing the overlap between our dataset of common differentially expressed genes (ICM versus ERSE or APE) and the differentially expressed genes between 2i-mESCs and serum-mESCs or serum-mESCs and mEpiSCs. **D** Dot plots showing the expression values of newly discovered pluripotency-associated genes. I: ICM; E: ERSE; A: APE. **E** RT-qPCR showing the expression levels of 3 newly discovered pluripotency-associated genes in mouse isolated ICM, whole blastocyst, ERSE and APE. Expression values are normalized against *Gapdh*. 3 independent biological replicates were analysed. Error bars represent the standard deviation. **F** Immunocytochemistry showing the expression of Klf4 and Oct4 in mouse blastocysts and ERSE stage embryos. DAPI staining is shown in blue. Left panel indexed with a “P” represent the maximum projection of the z-stacks for the ERSE embryos. 3-4 independent embryos were analysed and these are representative embryos.

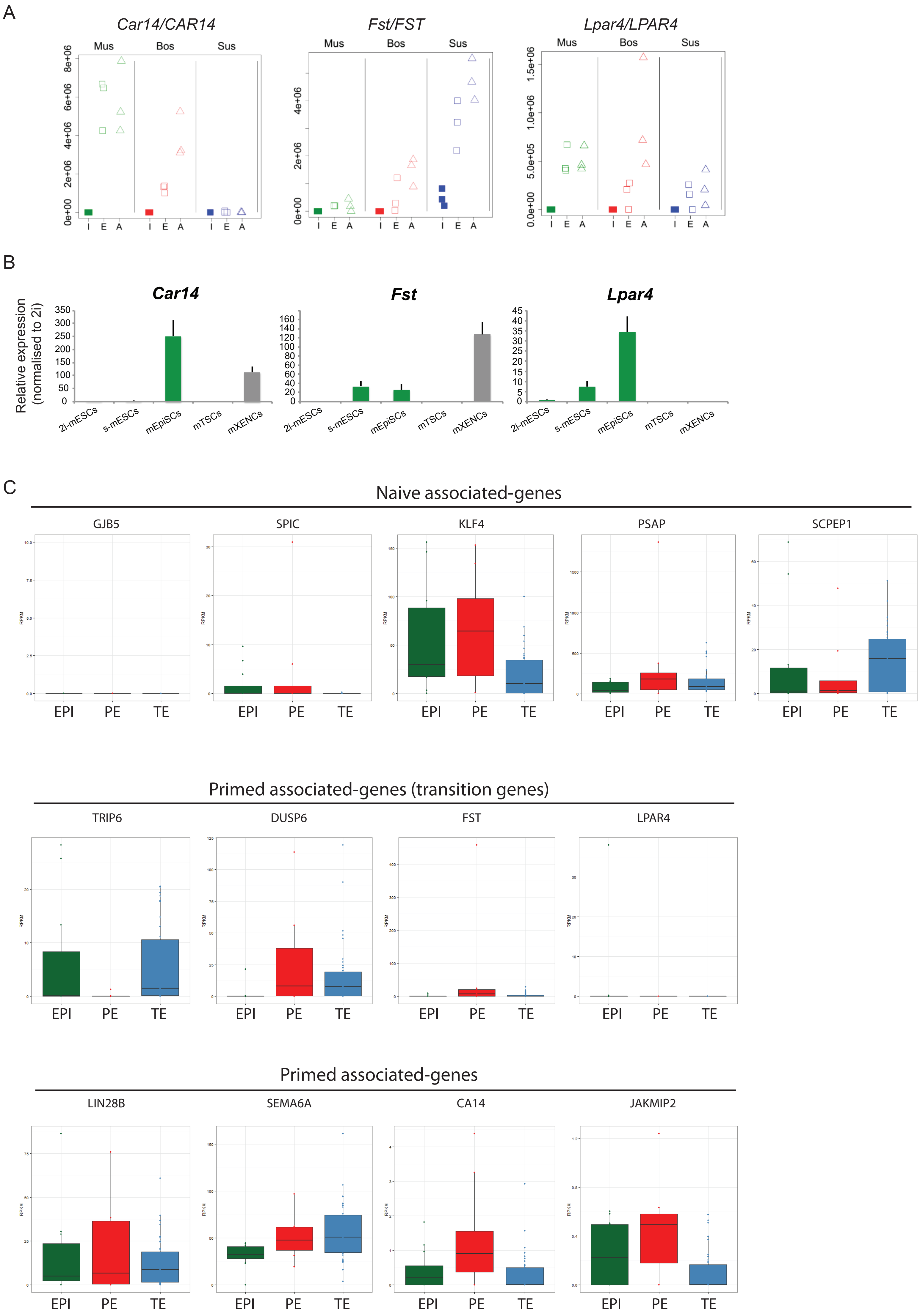

Biology Open • Supplementary information

Supplementary Figure 6 related to Figure 6: A Dot plots showing the expression values of 3 other identified pluripotency-associated genes that we included in our analysis. I: ICM; E: ERSE; A: APE. **B** RT-qPCR showing the expression levels of the genes shown in panel (A) in mouse stem cells (2i-mESCs or serum-mESCs; mEpiSCs; mTSCs; mXENCs). Expression values are normalized against *Gapdh*. 3 independent biological replicates were analysed. Error bars represent the standard deviation. **C** Box plots showing the expression values of cells within the pre-implantation human blastocyst as indicated (EPI, epiblast; PE, primitive endoderm; TE, trophectoderm). Data was directly collated from Blakeley et al. 2015.

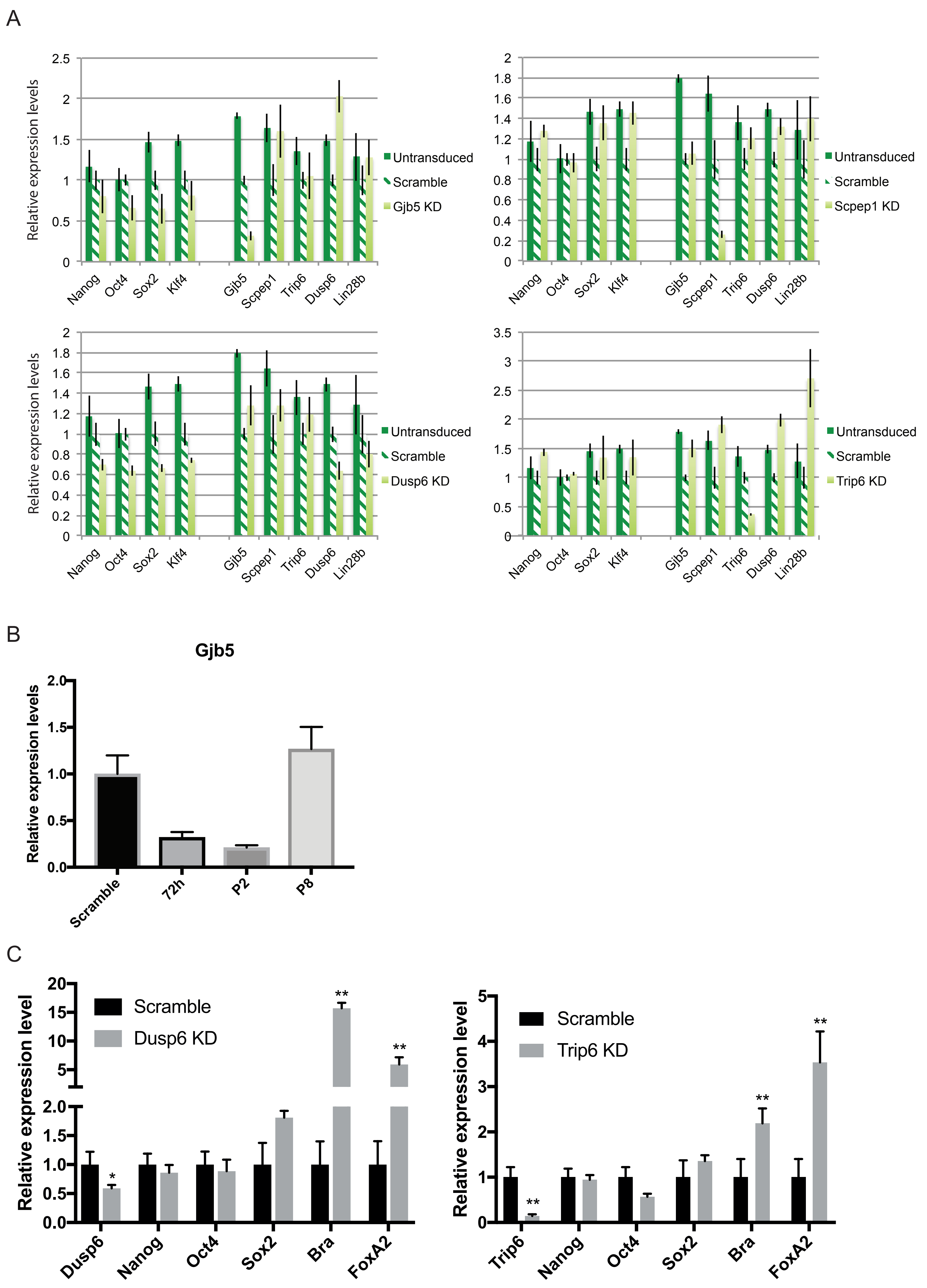

Biology Open • Supplementary information

Supplementary Figure 7 related to Figure 7: A RT-qPCR showing the expression levels of core pluripotency genes and newly discovered pluripotency-associated genes in knockdown, untransduced and control scramble transduced mESCs 72h after transduction. Expression values are normalized against *Gapdh*. 3 independent biological replicates were analysed. Error bars represent the standard deviation. **B** RT-qPCR showing the expression levels of *Gjb5* in knockdown and control scramble transduced mESCs 72h after transduction, 2 passages (P2) after transduction and 8 passages (P8) after transduction. Expression values are normalized against *Gapdh*. 3 independent biological replicates were analysed. **C** RT-qPCR showing the expression levels of core pluripotency genes, differentiation genes and the newly discovered pluripotency-associated genes *Dusp6* or *Trip6* in knockdown and control scramble transduced mEpiSCs 72h after transduction. Expression values are normalized against *Gapdh*. 3 independent biological replicates were analysed. * represents a p value of 0.05 or less; ** represents a p value of 0.01 or less.

II. Supplemental Tables

Table S1 Summary of all samples analysed in this study and their respective GEO number.

Click here to Download Table S1

Table S2 Dataset of expression values of all 1:1:1 orthologs.

Click here to Download Table S2

Table S3A KEGG pathways significantly enriched (minimum adjusted (Benjamini) Pvalue 1%) among conserved genes expressed at each stage. The 3 pathways highlighted with a star are the only ones specific to the APE stage.

Click here to Download Table S3A

Table S3B Dataset of expression values for known pluripotency genes and components of pathways and of the epigenetic machinery involved in pluripotency in ICM and ERSE stage mouse, bovine and porcine embryos.

Click here to Download Table S3B

Table S4A Differentially expressed genes between ICM and ERSE, ICM and APE as well as ERSE and APE in the three species.

Click here to Download Table S4A

Table S4B Lists of genes contained in the territories of the Venn diagrams of Fig. 4A and S4A.

Click here to Download Table S4B

Table S5A Differentially expressed genes between: 1) 2i-mESC and serum-mESCs; 2) serum-mESCs and mEpiSCs; and 3) 2i-mESCs and mEpiSCs.

Click here to Download Table S5A

Table S5B Lists of genes contained in the territories of the Venn diagrams of Fig. 5B and S5C.

Click here to Download Table S5B

Biology Open • Supplementary information

III. Supplemental Experimental Procedures

1. Cell culture (ESC, EpiSC)

1.1 Mouse cell lines

C57Bl/6xDBA/Ja (mESCs 1) and 129S2/SvPas (mESCs 2) mESCs were cultured on γ-irradiated primary mouse embryonic fibroblasts (MEF; CF1 derived fibroblasts) in conventional ESC growing media [high glucose Dulbecco-modified Eagle medium (DMEM, Invitrogen, Carlsbad, USA), supplemented with 1000 IU/ml recombinant mouse leukaemia inhibitory factor (ESGRO-LIF, Chemicon-Millipore, Billerica, USA), 15% fetal bovine serum (FBS, HyClone, Thermo Fisher Scientific, Waltham, USA), 1x nonessential amino acids (NEAA, Invitrogen), 0.2 mM 2-mercaptoethanol (Invitrogen), 50 IU penicillin/ml (Invitrogen), 50 mg streptomycin/ml (Invitrogen)]. Alternatively, they were grown feeder free in 2i + LIF containing medium as described in (Nichols and Smith, 2009). The culture dishes were kept at 37°C in a humidified atmosphere of 5% CO2 in air. C57Bl/6xDBA/Ja (mEpSCs 1) and 129S2/SvPas (mEpiSCs 2) mEpiSC were cultured on gelatine and MEF-medium coated plates in chemically defined medium (CDM) with 12 ng/ml FGF2 (R&D systems, Minneapolis, USA) and 20 ng/ml Activin A (R&D systems, Minneapolis, USA). They were routinely passaged as small clumps using Collagenase II (Sigma-Aldrich, St-Louis, USA). The culture dishes were kept at 37°C in a humidified atmosphere of 5% CO2 in air.

1.2 Transduction experiments

After detachment and disaggregation with accutase (Sigma), 2i-mESCs were seeded at a density of 5 x 104 cells/12 well plate well two days before transduction with lentivirus. The cells then received fresh culture medium and an aliquot of each of two different shRNA-expressing lentiviruses. 72hr after lentiviral transduction, cells were imaged on an LSM710

Biology Open • Supplementary information

Zeiss confocal microscope to determine GFP expression and therefore assess for transduction efficiency. Next, cells were either collected in Trizol reagent for analysis of gene expression by qPCR, or dissociated with PBS-based cell-dissociation buffer (Life Technologies) for flow cytometric analysis. Some of the cells for flow cytometry were stained immediately after harvest with anti-SSEA-1-PE (BD Pharmingen) (clone MC480), then fixed using a kit component from the Cytofix/Cytoperm kit (BD Biosciences). The remainder were fixed, permeabilized and stained with anti-Oct3/4-BV510 (BD Biosciences, clone 40/Oct3), anti-Nanog-AF647 (BD Biosciences, clone M55-312) and rabbit polyclonal anti-Sox2 antibody (Millipore) followed by donkey-anti-rabbit-AF594 (e-bioscience) secondary antibody.

2. Embryo sample preparation

Not all embryos develop at the exact same rate, and this could be a confounding factor in our experiment. As such, 3-5 embryos were pooled per sample and 3 samples were collected per stage and per species for further analysis.

2.1 Animals

The animal experiments were established in full compliance with European and National laws and regulations.

2.2 Preimplantation stage mouse embryo sample preparation

2.2.1 Blastocyst collection

6–12 weeks old C57Bl/6J (Harlan Laboratories, Indianapolis, USA) female mice were superovulated by injection with 5 IU pregnant mare serum gonadotropin (PMSG; Folligon®, Intervet International B.V., Boxmeer, The Netherlands) followed by injection with 5 IU human chorionic gonadotropin (hCG; Choragon®, Richter Gedeon Rt., Budapest, Hungary) 48 hrs later. Females were mated with DBA/2J genotype males (Harlan Laboratories) to

Biology Open • Supplementary information

produce F1 (C57Bl/6J x DBA/2J) embryos. Blastocyst stage embryos were collected at E3.5 from the uterus of female mice by flushing with with Chatot-Ziomek-Bavister medium (CZB-Hepes) (Chatot et al., 1989). Blastocysts were kept in a microdrop of KSOM (KSOM+AA; EmbryoMax, Millipore-Merck) under mineral oil (Sigma-Aldrich) at 37°C with 5% CO2 and 90% humidity until further manipulation.

2.2.2 Laser assisted ICM isolation

Laser-assisted (XY Clone, Hamilton Thorne, UK) ICM isolation was performed on intact, expanded E3.5 blastocysts as previously described (Tanaka et al., 2006). In sum, the embryos were secured by two holding pipettes with the ICM being positioned at 9 o'clock (Figure S1). Once adequate tension was established, several (about 10-15) infrared laser pulses (300mW, 1ms) were fired to section the blastocyst into two uneven portions: one containing the ICM and few trophectoderm cells, while the other contained only TE cells.

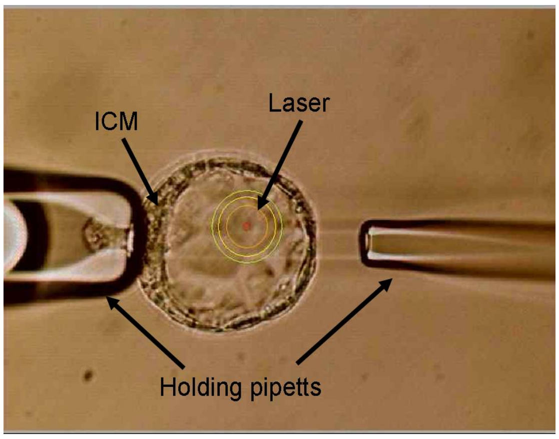

Figure S1. ICM isolation by laser.

2.2.3 Immunosurgery

ICMs were isolated from E3.5 F1 (B6D2) blastocysts by immunosurgery (Figure S2.) (Solter and Knowles, 1975). After removal of the zona pellucida by incubation in acid Tyrode's solution (Sigma-Aldrich), blastocysts were placed in KSOM media containing 20% anti-mouse

Biology Open • Supplementary information

serum (Sigma-Aldrich) for 30 min in 37°C humidified incubator. After 3 times washing in KSOM, blastocysts were incubated for 5-10 min in guinea pig complement (Calbiochem-Merck) diluted 1:10 with KSOM. After serial washings, the embryos were cultured further 30-60 min in KSOM. Finally, the ICMs were pipetted through the thin micropipette, in order to mechanically remove the lysed TE cells.

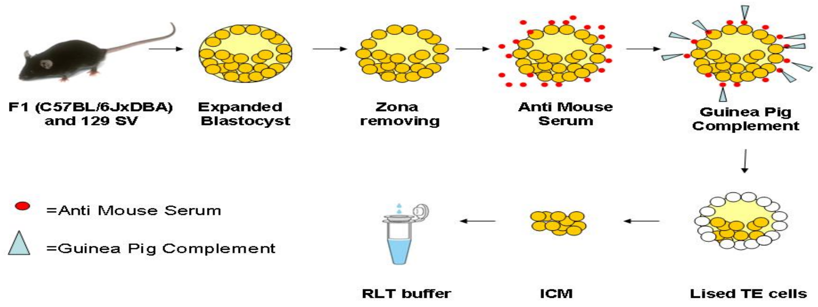

Figure S2. The procedure of immunosurgery on mouse blastocyst

2.3 Post-implantation mouse embryo epiblast isolation

Post-implantation stage embryos were dissected as described previously (Nagy, 2003). In detail: implanted epiblast stage mouse embryos were dissected at two time points: at pre-gastrulation stage (ERSE stage) and at mid-gastrulation stage (APE stage). ERSE samples were collected at E6.25 in the case of F1 mice and at E6.75 in the case of 129S2 mice. APE samples were collected at E6.75 in the case of F1 mice and at E7.25 in the case of 129S2 mice. Pregnant female animals were euthanized by cervical dislocation. After surgical removal of the uterine horns, the uterus was placed in PBS (Sigma-Aldrich). Embryos were sectioned out from the decidua first, by using sharp fine edge forceps. After removing the embryo from the decidua the Reichart's membrane was tear out from the embryo and the ectoplacental cone (trophoblast) tissue was sliced out. The visceral endoderm and other extraembryonic

Biology Open • Supplementary information

tissues were removed from both ERSE and APE embryos and while the ERSE stage epiblast was collected as clean epiblast, the APE stage epiblast was collected with its attached mesodermal wings.

2.4 Pig embryo sample preparation

Uterine horns from slaughtered sows were flushed with 150 ml embryo flushing medium (PBS with 1% FBS; Sigma-Aldrich) at 7, 10.5 and 12.5 days after insemination. Embryos (Danish Landrace x Yorkshire crosses) were collected by filtration of flushing fluid trough a filter and embryos were collected under a stereomicroscope (for E7 and 12.5). At E10.5, embryos were not flushed the uterine horns were placed in a tub with embryo flushing media and clipped open. The uterus was left in the tub for 15 min and during this time embryos floated to the surface and were collected with a bulb pipette. In filamentous embryos the embryonic disc and the nearest trophectoderm was carefully separated from the other embryos using forceps and the main part of the trophectoderm was cut off with a scalpel thereby releasing the embryo from the entangled trophectoderm.

2.5 Bovine embryo sample preparation

Bovine ovaries were recovered at the abattoir from Holstein cows subjected to routine veterinary inspection and in accordance to the specific health requirements stated in Council Directive 89/556/ECC and subsequent modifications. The ovaries were placed in an insulated container in sterile PBS and were transferred to the laboratory in a warm box kept at 25 °C. Oocytes were recovered by aspiration, were selected for non-atretic cumulus cells morphology, and were matured for 20 to 24 h at 38.5°C in 5%CO2 and 5%O2 in medium TCM199 supplemented with 10% foetal calf serum (FCS, Invitrogen), gonadotropins (FSH and LH, 0.05 IU each; Menogon, Ferring, Italy), ITS (insulin, transferrin, sodium selenite, Sigma-Aldrich), Long-IGFI (100ng/ml, Sigma-Aldrich) and Long-EGF (50ng/ml, Sigma-Aldrich). For in

Biology Open • Supplementary information

vitro fertilization (IVF) one straw of commercially available frozen semen of a single Holstein bulls was thawed and the semen was separated on a Redigrad gradient (90%-45%) (Amersham Biosciences AB, Sweden). The pellet containing the motile fraction was counted and 350.000 sperm were added to 1ml of SOF medium (Synthetic Oviductal Fluid) (Gardner et al., 1994), supplemented with heparin (1µg/ml, Sigma-Aldrich) and sperm motility factors solution (PHE; 20 M D-penicillamine, 100 M hypotaurine, 1 M epinephrine; Sigma-Aldrich), according to Bavister (Bavister, 1989) and without MEM amino acids. Matured oocytes were co-incubated with the semen for 18 to 20 h at 38.5°C in 5%CO2 and 5%O2. The day after IVF (d 1) presumptive zygotes were transferred in SOF medium supplemented with Eagle MEM amino acids (Invitrogen) and BSA (4mg/ml, Sigma-Aldrich) until day 8 performing 50% changes of medium at day 4 and 6 at the same atmosphere as above. Expanded blastocysts with a pronounced inner cell mass (ICM) were selected on day 7-8 and were processed by immunosurgery as described previously for mouse (Solter and Knowles, 1975). For later stages (ERSE and APE), donor cows were superovulated and bred after induced estrus (day0). Day 12 to 18 embryos were collected by nonsurgical flushing in warm flushing medium (FHM). Epiblast was then removed from the surrounding trophoblast and underlying hypoblast using glass needles under a stereomicroscope.

3. Immunohistochemistry (IHC)

mESCs and mEpiSCs grown on gelatine coated round cover slips, and mouse embryos were fixed in 4% PFA fixative at RT, for 15-30 min, followed by three-times washing steps in PBS containing 1% BSA and 0.1% Triton X-100 (Sigma-Aldrich) for 10 min. For blocking, washing solution containing 10% FBS was used for 1 hour. Primary antibodies (Table SM1) were incubated in blocking solution 2.5 hours at RT, in the indicated dilution. After three washes the samples were incubated at RT for 1h with the host matching secondary antibody in the indicated dilution (Table S1). Samples were mounted with Vectashield-DAPI mounting media

Biology Open • Supplementary information

(Vector Laboratories) or with ProLong Gold antifade reagent with DAPI (Invitrogen), thus the nuclei were labelled with DAPI (blue). The immunostainings were visualized with an AxioObserver Z.1 inverted fluorescent microscope imaged using the ApoTome slider system and AxioCam MRm camera system (Carl Zeiss GmbH). Alternatively, immunostainings were visualised and imaged using a Zeiss Lsm 710 microscope.

4. Flow cytometry

mESCs were detached and disaggregated with PBS-based enzyme-free cell-dissociation buffer (Life Technologies). After filtration through a 70µm mesh, the cells were washed in cold staining buffer (PBS with 1% Bovine Serum Albumin and 0.1% Sodium Azide) and counted on an EVE automated cell counter (NanoEntek). Antibodies used are detailed in Table SM2. For SSEA-1 staining, 5x105 cells were incubated with 30 ng PE-conjugated anti-SSEA-1 (clone MC480, BD Pharmingen) or mouse IgMk isotype control (clone MM-30, BioLegend) in a 100 µl volume for 30 min on ice. They were then washed twice in cold staining buffer, fixed for 20 min on ice using Cytofix/Cytoperm reagent (BD Biosciences), washed, and resuspended in staining buffer ready for analysis. For transcription factor staining, 5x105 cells were fixed and permeabilised with Cytofix/Cytoperm according to the manufacturers' recommended protocol. The cells were stained in a 100 µl volume with the following antibodies: 250 ng AlexaFluor647-conjugated anti-Nanog (clone M55-312, BD Pharmingen) or IgG1k isotype control (clone MOPC-21, BioLegend); 1 µg Brilliant Violet 510-conjugated anti-Oct3/4 (clone 40/Oct-3, BD Biosciences) or IgG1k isotype control (clone MOPC-21, BioLegend); 1 µg unconjugated rabbit polyclonal anti-Sox2 (Millipore, Catalogue number AB5603) or rabbit IgG isotype control (R & D Systems), followed by 1 µg AlexaFluor647-conjugated Donkey-anti-Rabbit secondary antibody (Life Technologies). Cells were analysed using a BD LSR Fortessa X20 cytometer. Cell doublets and debris were excluded from analysis using forward/side scatter gating parameters in FlowJo v10.0.7

Biology Open • Supplementary information

(Treestar) and 10,000 or more appropriately compensated events were plotted as modular (normalised) histograms with smoothing applied.

5. In situ Hybridization (ISH)

For whole-mount ISH, embryos were rehydrated, washed in PBST (0.01M Phosphate buffer saline pH 7.4, 0.1% Tween 20; Sigma-Aldrich) and embryos were subjected to proteinase K (10µg/ml in PBST, Invitrogen) digestion for 10 min. Embryos were fixed again in 4% paraformaldehyde/ 0.2% glutaraldehyde and washed. Hybridization with Dig-labeled riboprobes was performed as described (Weisheit et al., 2002). After incubation with anti-Dig antibody, embryos were incubated in BM purple (Roche) until the colour developed. All processed samples were photographed under a microscope or a stereomicroscope using a digital camera (Olympus). The bovine antisense RNA probes OCT4, NANOG, SOX2 and T were generated as described (Degrelle et al., 2005; Hue et al., 2001) and corresponded to NCBI entries: DQ126156, DQ126153.1, DQ126150 and NM_001192985.1, respectively. They were used also for pig embryo hybridization due to the high conservation with pig cDNA sequence (minimum 87%). For mouse embryos, probes were obtained as follows: Oct4, from A. Smith; T, from B. Herrmann (Herrmann et al., 1990); Nanog and Sox2, from J. Rossant. The hybridized embryos were observed under an inverted microscope and photographed using a digital camera (Zeiss).

6. mRNA preparation

6.1 From embryo tissues

RNA was isolated using TriZol (Life Technologies) and the PureLink™ RNA Micro Kit (Invitrogen), following the manufacturer’s instructions. In short, samples were collected in TriZol and homogenised by pipetting up and down following by 5 min incubation at 30 °C.

Biology Open • Supplementary information

RNA was isolated in chloroform and precipitated in 70% ethanol. Precipitated RNA in ethanol was transferred to a PureLink RNA micro kit spin cartridge and further purified according to the manufacturer instructions. Integrity of the RNA samples was confirmed using the Experion Automated Electrophoresis Station (Bio-Rad Laboratories, Hercules, USA). Concentration of the RNA samples was determined by a Qubit fluorometer (Invitrogen) and the Experion.

6.2 From cells

RNA was isolated using TriZol (Life Technologies), following the manufacturer's instructions. In short, samples were collected in TriZol and homogenised by pipetting up and down following by 5 min incubation at 30 °C. RNA was isolated in chloroform and precipitated in 70% ethanol. Precipitated RNA in ethanol was dissolved in DNase-free water. RNA sample purity and quantification was determined using a NanoDrop instrument.

7. cDNA library preparation and sequencing

In order to generate RNA-Seq expression profiles from the early embryonic stages, the Ovation RNASeq kit (NuGen, San Carlos, USA), the SMARTer Ultra Low RNA kit from Illumina (San Diego, USA) and an adaptation of the Kurimoto et al. protocol (Kurimoto et al., 2007) were tested for low quantity RNA-Seq expression profiling on mouse liver RNA. The results were compared to regular RNA-Seq profiling. Regular RNA-Seq profiling was performed on 100 ng of RNA, using the RiboZero kit (EpiBio, Medison USA) to deplete for ribosomal RNA, and hexamers for priming the reverse transcription, followed by regular sample preparation for sequencing.

7.1 Ovation RNA-Seq System (Ribo-SPIA® Technology)

Biology Open • Supplementary information

100 pg of mouse liver total RNA was amplified using the Ovation RNA-Seq kit (7100-08, NuGen). cDNA was sonicated using the Bioruptor (Diagenode, Liege, Belgium) for 15 min (30 sec on, 30 sec off, high power). Paired-end sequence adaptors were ligated to DNA fragments, followed by size selection (300 bp; E-Gel® Agarose Gel Electrophoresis System of Invitrogen) and 14 cycles of PCR amplification.

7.2 SMARTer Ultra Low RNA kit from Illumina

For all samples, 100 pg of RNA was used for cDNA synthesis and amplification using the SMARTer™ Ultra Low RNA Kit for Illumina Sequencing (Clontech; Cat. No. 634935) using 20 PCR cycles. The amplified cDNA was fragmented by sonication (Bioruptor, Diagenode). Paired-end sequence adaptors were ligated to DNA fragments, followed by size selection (300 bp; E-Gel® Agarose Gel Electrophoresis System of Invitrogen) and 14 cycles of PCR amplification.

7.3 Kurimoto protocol for small sample amplification

100 pg of mouse liver total RNA were amplified using an adapted version of the Kurimoto protocol. The main changes to their published protocol included the use of an increased amount of superscript III, primers and dNTPs as well as a longer RT reaction. The amplified cDNA was fragmented by sonication (Bioruptor, Diagenode). Paired-end sequence adaptors were ligated to DNA fragments, followed by size selection (300 bp; E-Gel® Agarose Gel Electrophoresis System of Invitrogen) and 14 cycles of PCR amplification.

7.4 Sequencing

Cluster generation and sequencing (2*100 bp) was performed with the Illumina HiSeq platform according to standard Illumina protocols. The first mate per pair was trimmed to 50 bp and aligned to the mouse mm9/NCBI37 (Jul. 2007), bosTau6/UMD3.1 (Nov. 2009) or

Biology Open • Supplementary information

Sscrofa 10.2 (Aug. 2011) assemblies using GSNAP (Version 2012-01-11; -N 1 -n 1 -Q --quality-protocol=sanger). The mapping used unique splice sites derived from the UCSC annotation (mm9: 372757 unique splicesites; susScr10.2 without annotation, no known splicesites; bosTau6 230347 unique splicesites). Only the tags aligning to one position on the genome were considered for further analysis. The output data were converted to Browser Extensible Data (BED) files for downstream analysis and Wiggle (WIG) files for viewing. All RNA-seq data (FASTQ, BED, and WIG files) are present in the NCBI GEO SuperSeries GSE53387. Summary of the sequencing output files can be found in Table SM3.

8. Bioinformatics analysis

8.1 Processing of RNA-seq data

To obtain expression values and differential expression calls, the uniquely mapping reads were used as input to cufflinks and cuffdiff (Trapnell et al., 2010) (Version 1.3.0) (parameters: max-bundlefrags=10000000, compatible-hits-norm, num-importance-samples=5000, upper-quartile-norm, bootstrap-fraction=1.0, num-bootstrap-samples=100, max-mle-iterations=10000). Given that UTRs were mostly unannotated in pig and cow, we quantified expression based on the coding regions annotated in the Ensembl database (Hubbard et al., 2009) (Version 67). The per-sample expression values were re-normalized using the method of Anders and Huber (Anders and Huber, 2010) derived from the genes that were among the highest 5% expressed in all samples. Principal component analysis was performed on the covariance matrix of the renormalized and square-root transformed expression values. Genes that could not be included in the cuffdiff 1.3.0 analysis were analyzed by the updated version cuffdiff 2.1.1 (marked in Table S4A with “CD2”; parameters: max-bundlefrags=10000000, compatible-hits-norm, dispersion-method=pooled, library-normmethod=geometric, num-fragment-count-draws=1000, num-frag-assign-draws=150, max-mleiterations=15000).

Biology Open • Supplementary information

8.2 Orthology relations

The orthology relations from Ensembl (Hubbard et al., 2009) (Version 67) were downloaded from Ensemble biomart (Kinsella et al., 2011) using the R package biomaRt (Durinck et al.). For the interspecies comparisons we only considered the gene pairs marked as 'ortholog_one2one' without alternative candidate orthologs ('apparent_ortholog_one2one' or 'possible_ortholog_one2one').

8.3 Conservation differential expression

In order to calculate how much evolutionary conservation there was in differentially expressed genes between stages, we first calculated the numbers of differentially expressed genes (q=0.25) per species between any two stages, only considering 1:1:1 orthologs (11212 genes), and only considering either an increase in gene expression or a decrease in gene expression. We then calculated the expected overlap in the absence of conservation by multiplying these numbers with each other for two species and dividing the resulting number by the number of 1:1:1 orthologs. The observed number of conserved, differentially expressed 1:1:1 orthologs between two species was then divided by the corresponding expected number to obtain a fold change in conserved differentially expressed genes. To give an example, 120 genes are "down" from ICM to ERSE in both M. musculus and B. taurus, while the number of genes that would be expected to be "down" in both species if there were no conservation is 65. The latter number is the product of the numbers of 1-1-1 orthologs "down" in either species divided by the total number of 1-1-1 orthologs, e.g. 885 (down in B. taurus) * 824 (down in M. musculus) / 11212 = 65. The conservation of differential expression thus is 120/65=1.85. For the conservation of differential expression between three species, the expected overlap is the product of the three numbers of differentially expressed genes divided by the square of the number of 1:1:1 orthologs. As with the conservation between two species, the observed overlap of differential expression

Biology Open • Supplementary information

among three species is then divided by the expected overlap to obtain a fold change in conserved differentially expressed genes. Statistical significance of the conservation of differential expression was calculated by randomizations: how often does one, when randomly selecting the same numbers of genes that show differential expression per species, observe an overlap in differential expression between species that is at least as large as the observed overlap. In principle, one could also calculate the significance of the overlap analytically using the hypergeometric distribution, but this becomes rather cumbersome for large numbers. Analytical calculations using the binomial distribution, that approximates the hypergeometric distribution for large numbers, gave similar significance values as observed by the randomizations.

8.4 Enrichment and depletion analysis of differentially expressed genes shared between in vitro and in vivo datasets

This enrichment/depletion was calculated analogously to the conservation of differential expression between species, i.e the overlap in genes that are both higher(lower) expressed from one in vitro stage to another and from one in vivo stage to another was divided by the expected overlap. The expected overlap is the product of: 1) the number of genes that are higher(lower) expressed from one in vitro stage to another, and 2) the number of genes that are higher(lower) expressed from one in vivo stage to another, divided by the total number of 1:1:1 orthologs. The observed overlap divided by the expected overlap can give both an enrichment (when it is higher than 1) or a depletion (when it is lower than 1). The significance was calculated by randomizations (hyper geometric distribution), analytical calculations for a binomial distribution gave similar significance values. The in vivo/in vitro overlaps were calculated for 1:1:1 orthologs to allow comparison of the enrichment in mouse only or with the genes conserved differentially expressed among the three species.

Biology Open • Supplementary information

9. Quantitative Real-time PCR analysis

9.1 cDNA synthesis

For RT-qPCRs the total RNA was isolated from 3 biological replicates of mESCs and mEpiSCs using RNeasy Plus Mini Kit (Qiagen, Hilden, Germany). For RNA isolation from mouse samples pools of 20 B6D2 isolated ICMs, blastocysts, or of 10 ERSE or APE embryos, the RNeasy Plus Micro Kit (Qiagen) were used. 0.5 µg of mESC and mEpiSC RNA and 0.3 µg RNA from the ICM, blastocysts, ERSE and APE samples were used for reverse transcription. For RNA isolation from bovine samples pools of 40 blastocysts, of 3 ERSE and 1 APE, the PureLink™ RNA Micro Kit (Invitrogen) was used. 20 to 30 ng of total RNA was used for reverse transcription. For RNA isolation from pig samples pools of 10 blastocysts, or of 8 ERSE or 6 APE embryos, extracted with the RNeasy Plus Micro Kit (Qiagen) were used. 20ng to 30 ng of total RNA was used for reverse transcription. cDNA was synthesised using the SuperScript III cDNA Synthesis Kit (Invitrogen) according to the manufacturer's instructions.

9.2 Primer design and RT-qPCR

Primers (Tables SM4, SM5 and SM6) for the selected genes were designed using Primer3 (Rozen and Skaletsky, 1999). Primers were optimized using two-fold serial dilution standard curves. Following primer optimization, 8 selected genes and the Gapdh refrence gene were used for real time PCR analysis as shown in Table S4. Each real time PCR reaction consisted of cDNA template, 200 nM of each primer and 50% SYBR Green Roche ReadyMix (Roche) in a total volume of 10 µl.. A Roche 480 light cycler or a StepOne Plus (Applied Biosystems) was used to perform the PCRs. The cycling parameters were as follows: 95°C for 5 min initial denaturation followed by 40 cycles of 95°C for 10 s, 60°C for 15 s and 72°C for 30 s. Melting curve analysis and agarose gel electrophoresis confirmed the specificity of the primers and

Biology Open • Supplementary information

the absence of gDNA contamination. Data of three biological replicates were analysed for each gene using excel.

10. Retroviruses engineering and production

10.1 Retrovirus construction

pLKO.1 retroviral plasmid constructs containing gene-specific shRNA sequences were purchased from Dharmacon (see supplementary methods for The RNAi Consortium (TRC) designations). These were digested with Acc65I and BamHI to remove the puromycin resistance cassette. Subsequently, the EGFP-IRES-Puromycin resistance (EGIP) cassette from pEGIP (Addgene plasmid #26777) (Zou et al., 2009) was amplified by PCR with the following primers: Forward- CTCCCCAGGGGGATCCACGCCGCCACCATGGTGAGCAAGGGCGAG and Reverse- TGGTCTTAAAGGTACCTCAGGCACCGGGCTTGCGGGTCATGCACCA using KAPA HiFi (Kapa Biosystems). The amplified EGIP cassette was inserted into the linearized pLKO.1 plasmids using the InFusion system (Clontech), preserving the BamHI and Acc65I restriction sites.

10.2 Retrovirus production

Retroviral particles were produced according to the method detailed in Nature Methods (Kutner et al., 2009). Briefly, early passage 293T cells at 30-40% confluence were transfected with pMD2.G (Addgene #12259), psPAX2 (Addgene#12259) and the pLKO.1-shRNA-EGIP constructs in a ratio of 4.5:9: 18µg plasmid DNA, and 1: 2.5µg DNA:µg polyethylimine. Viral particles were collected from the supernatant 24 and 48 hours after transfection. Upon collection, supernatants were filtered through 0.45µm pore syringe filters and excess plasmid DNA was removed by adding [1mM] MgCl2 and 5U/ml DNAse I (Roche Diagnostics) and incubating at room temperature for 20 minutes. Supernatants were centrifuged over a cushion of 20% (w/v) sucrose for 2 hours at an average RCF of 61,071, at 4°C. The pellet of

Biology Open • Supplementary information

viral particles was resuspended in CDM medium, aliquotted and snap-frozen on dry-ice, before being stored at -80°.

Biology Open • Supplementary information

IV. Supplemental References

Anders, S., and Huber, W. (2010). Differential expression analysis for sequence count data. Genome Biol *11*, R106.

Bavister, B.D. (1989). A consistently successful procedure for in vitro fertilization of golden hamster eggs. Gamete research *23*, 139-158.

Chatot, C.L., Ziomek, C.A., Bavister, B.D., Lewis, J.L., and Torres, I. (1989). An improved culture medium supports development of random-bred 1-cell mouse embryos in vitro. J Reprod Fertil *86*, 679-688.

Degrelle, S.A., Campion, E., Cabau, C., Piumi, F., Reinaud, P., Richard, C., Renard, J.P., and Hue, I. (2005). Molecular evidence for a critical period in mural trophoblast development in bovine blastocysts. Dev Biol *288*, 448-460.

Durinck, S., W. Huber and S. Davis biomaRt: Interface to BioMart databases (e.g. Ensembl, Wormbase and Gramene).

Gardner, D.K., Lane, M., Spitzer, A., and Batt, P.A. (1994). Enhanced rates of cleavage and development for sheep zygotes cultured to the blastocyst stage in vitro in the absence of serum and somatic cells: amino acids, vitamins, and culturing embryos in groups stimulate development. Biol Reprod *50*, 390-400.

Herrmann, B.G., Labeit, S., Poustka, A., King, T.R., and Lehrach, H. (1990). Cloning of the T gene required in mesoderm formation in the mouse. Nature *343*, 617-622.

Hubbard, T.J., Aken, B.L., Ayling, S., Ballester, B., Beal, K., Bragin, E., Brent, S., Chen, Y., Clapham, P., Clarke, L.*, et al.* (2009). Ensembl 2009. Nucleic Acids Res *37*, D690-697.

Hue, I., Renard, J.P., and Viebahn, C. (2001). Brachyury is expressed in gastrulating bovine embryos well ahead of implantation. Development genes and evolution *211*, 157-159.

Kinsella, R.J., Kahari, A., Haider, S., Zamora, J., Proctor, G., Spudich, G., Almeida-King, J., Staines, D., Derwent, P., Kerhornou, A.*, et al.* (2011). Ensembl BioMarts: a hub for data retrieval across taxonomic space. Database : the journal of biological databases and curation *2011*, bar030.

Kurimoto, K., Yabuta, Y., Ohinata, Y., and Saitou, M. (2007). Global single-cell cDNA amplification to provide a template for representative high-density oligonucleotide microarray analysis. Nat Protoc *2*, 739-752.

Kutner, R.H, Zhang, X-Y, and Reiser, J. (2009). Production, concentration and titration of pseudotyped HIV-1-based lentiviral vectors. Nat Protoc *4*, 495-505.

Biology Open • Supplementary information

Nagy, A. (2003). Manipulating the mouse embryo: a laboratory manual. Cold Spring Harbor, N.Y., Cold Spring Harbor Laboratory Press.

Nichols, J., and Smith, A. (2009). Naive and primed pluripotent states. Cell Stem Cell *4*, 487-492.

Rozen, S. and H. Skaletsky (1999). Primer3 on the WWW for General Users and for Biologist Programmers. Bioinformatics Methods and Protocols SE - 20. S. Misener and S. D.-.-.-.-. Krawetz, Humana Press DA - 1999/01/01**:** 365-386 LA - English.

Solter, D., and Knowles, B.B. (1975). Immunosurgery of mouse blastocyst. Proc Natl Acad Sci U S A *72*, 5099-5102.

Tanaka, N., Takeuchi, T., Neri, Q.V., Sills, E.S., and Palermo, G.D. (2006). Laser-assisted blastocyst dissection and subsequent cultivation of embryonic stem cells in a serum/cell free culture system: applications and preliminary results in a murine model. J Transl Med *4*, 20.

Trapnell, C., Williams, B.A., Pertea, G., Mortazavi, A., Kwan, G., van Baren, M.J., Salzberg, S.L., Wold, B.J., and Pachter, L. (2010). Transcript assembly and quantification by RNA-Seq reveals unannotated transcripts and isoform switching during cell differentiation. Nat Biotechnol *28*, 511-515.

Weisheit, G., Mertz, D., Schilling, K., and Viebahn, C. (2002). An efficient in situ hybridization protocol for multiple tissue sections and probes on miniaturized slides. Development genes and evolution *212*, 403-406.

Zou J, Maeder ML, Mali P, Pruett-Miller SM, Thibodeau-Beganny S, Chou BK, Chen G, Ye Z, Park IH, Daley GQ, Porteus MH, Joung JK, Cheng L. (2009). Gene targeting of a disease-related gene in human induced pluripotent stem and embryonic stem cells. Cell Stem Cell. 2009 *5,* 97-110.

Biology Open • Supplementary information

V. Supplemental Tables

Table SM1 Antibodies used in the immunostaining experiments

Table SM2 Antibodies and controls used in the flow cytometry experiments

Table SM3 Summary of sequencing output

Table SM4 Mouse RT-qPCR primer sequences used in the study

Table SM5 Bovine RT-qPCR primer sequences used in the study

Table SM6 Pig RT-qPCR primer sequences used in the study

Biology Open • Supplementary information

Table SM1 Antibodies used in the immunostaining experiments

1st Ab	CatN (Supplier)	Dilution	2nd Ab	CatN (Supplier)	Dilution
**Oct-3/4 (N19)**	sc-8628 (SC)	1:400	Alexa Fluor® 594 Rabbit Anti-Goat IgG	A-11080 (MP)	1:2000
**Oct3/4 (H134)**	sc-9081 (SC)	1:400	Alexa Fluor® 594 Goat Anti-Rabbit IgG	A-11012 (MP)	1:2000
**Scpep1 (N3C3)**	GTX103381 (GT)	1:300	Alexa Fluor® 488 Goat Anti-Rabbit IgG	A-11008 (MP)	1:2000
**Klf4**	09-821 (MM)	1:500	Alexa Fluor® 488 Goat Anti-Rabbit IgG	A-11008 (MP)	1:2000
**Trip6**	GTX111504 (GT)	1:500	Alexa Fluor® 488 Goat Anti-Rabbit IgG	A-11008 (MP)	1:2000
**Lin28**	ab63740 (AB)	1:500	Alexa Fluor® 488 Goat Anti-Rabbit IgG	A-11008 (MP)	1:2000
**Sema6a**	AF 1615 (RD)	1:200	Alexa Fluor® 488 Rabbit Anti-Goat IgG	A-11078 (MP)	1:2000
**Follistatin**	sc-30194 (SC)	1:200	Alexa Fluor® 488 Goat Anti-Rabbit IgG	A-11008 (MP)	1:2000
**Gjb5**	NBP1-84333 (NB)	1:300	Alexa Fluor® 488 Goat Anti-Rabbit IgG	A-11008 (MP)	1:2000

Ab, Antibody; AB, Abcam; GT, GeneTex; RD, R&D Systems; SC, Santa Cruz; MP, Molecular Probes; NB, Novus Biologicals, MM, Merck-Millipore

Table SM2 Antibodies and controls used in the flow cytometry experiments

1st Ab	CatN (Supplier)	Concentration	2nd Ab	CatN (Supplier)	Dilution
**SSEA-1-PE (MC480)**	560142 (BD)	300ng/ml			
**IgMκ 🎗PE (MM-30)**	401609 (BL)	300ng/ml			
**Nanog-AF647 (M55-312)**	560279 (BD)	2.5µg/ml			
**IgG1κ 🎗AF647 (MOPC-21)**	400130 (BL)	2.5µg/ml			
**Oct3/4 (40/Oct-3)-BV510**	563524 (BD)	10µg/ml			
**IgG1κ 🎗BV510 (MOPC-21)**	400172 (BL)	10µg/ml			
**Sox-2**	AB5603 (M)	10µg/ml	Alexa Fluor® 594 Donkey-Anti-Rabbit IgG	A-21207 (LT)	1:400
**Rabbit IgG**	ab-105-c (RD)	10µg/ml	Alexa Fluor® 594 Donkey-Anti-Rabbit IgG	A-21207 (LT)	1:400

Ab, Antibody; BD, BD Pharmingen; BL, BioLegend; LT, Life Technologies; M, Millipore; RD, R&D Systems

Biology Open • Supplementary information

Table SM3 Summary of sequencing output

Sample	Genome	Read-pairs (FASTQ)	SE50 (read1; SAM-BAM)	%mapped U
Plurisys_sample_9_F1_ICM_1	mm9	184047144	113121948	61.5
Plurisys_sample_2nd_9_F1_ICM_2	mm9	172747490	110593482	64.0
Plurisys_sample_3rd_9_F1_ICM_3	mm9	107015713	69375625	64.8
Plurisys_sample_1_F1_E6_7_1	mm9	217096749	114732323	52.8
Plurisys_sample_2nd_1_F1_E6_7_2	mm9	233046891	128585875	55.2
Plurisys_sample_2_F1_E6_2_1	mm9	208737139	126706559	60.7
plurisys_sample_2nd_2_F1_E6_2_2	mm9	223903633	131778084	58.9
Plurisys_sample_2nd_8_173_EBI_Sample_7	bosTau6	184154328	138053300	75.0
Plurisys_sample_3rd_8_131_EBI_Sample_5	bosTau6	214906567	167976410	78.2
Plurisys_sample_8_ERSEE_9	bosTau6	189760339	144988536	76.4
Plurisys_sample_3_7_ERSE_J14	bosTau6	208488312	138966974	66.7
Plurisys_sample_2nd_3_6_ERSE_J14	bosTau6	160301522	93979432	58.6
Plurisys_sample_3rd_3_12_ERSE_J14	bosTau6	188921122	128061245	67.8
Plurisys_sample_4_17_APE_J17	bosTau6	211862987	149276435	70.5
Plurisys_sample_2nd_4_18_APE_J17	bosTau6	217490961	137883455	63.4
Plurisys_sample_3rd_4_20_APE_J17	bosTau6	213447137	143500591	67.2
Plurisys_sample_2nd_7_APE	bosTau6	180342560	134824148	74.8
Plurisys_sample_5_EBI_1	susScr2	181610754	91217544	50.2
Plurisys_sample_3rd_5_EBI_5	susScr2	194253957	82205730	42.3
Plurisys_sample_2nd_5_EBI_6	susScr2	76412714	31294907	41.0
Plurisys_sample_6_ERSEE_9	susScr2	194738016	97573329	50.1
Plurisys_sample_2nd_6_2_ERSE_5	susScr2	211596478	105122083	49.7
Plurisys_sample_3rd_6_ERSE	susScr2	226286054	111994324	49.5
Plurisys_sample_7_AAPEplusPAPE_13	susScr2	177644229	95143158	53.6
Plurisys_sample_3rd_7_1_ape3	susScr2	196277801	106970407	54.5

Table SM4 Mouse RT-qPCR primer sequences used in the study

Symbol	Description	GenBank Accession	Fwd and Rvs primer (5' - 3')	PCR product (bp)
Pou5f1	POU domain, class 5, transcription factor 1	NM_013633.3	AGCCGACAACAATGAGAACC<br>TCTCCAGACTCCACCTCACA	110
Spic	Spi-C transcription factor (Spi-1/PU.1 related)	NM_011461.3	TGGAATGTCACCCACAGAGA<br>CTGTACGGATTGGTGGAAGC	100
Gj5b	Gap junction protein, beta 5	NM_010291.3	AGGAGCGGGTACTAAGGGA<br>ACACGGAAGACGAAGACCA	163
Klf4	Kruppel-like factor 4 (gut)	NM_010637.3	ACTCACACAGGCGAGAAACC<br>AAGGCCCTGTCACACTTCTG	140
Scpep1	serine carboxypeptidase 1	NM_029023.3	GATCTGTCTCGTGCGACTGTG<br>CCCATACTTCCTTGCCTTCG	104
Psap	Prosaposin	AF037437.1	AATCCCTTCCTTGCGACATA<br>AGAGTCAACCACCTCCTTGC	161
Trip6	Thyroid hormone receptor interactor 6	NM_011639.3	GCTGTTACAAGTGTGAGGAGTGT<br>TGGTGACAGTGGCTGAGAG	138
Jakmip2	Janus kinase and microtubule interacting protein 2	NM_001163637.1	TACGGAGGCTGATGGATGAAA<br>CTCTCTCTTCGGACTGCTCA	176
Dusp6	Dual specificity phosphatase 6	NM_026268.3	ATGCGGGCGAGTTCAAATAC<br>CTGATACCTGCCAAGCAATG	148
Lin28b	Lin-28 homolog B (C. elegans)	NM_001031772.2	CATGGGATTCGGATTCATCT<br>TGGCTCTCCTTCTTTCAAGC	127
Sema6a	Sema domain, transmembrane domain (TM), and cytoplasmic domain, (semaphorin) 6A	NM_018744.2	CACCTCTGCGTTCTCTTCCT<br>GAATGTGACTGTGGCCTGTG	137
Fst	Follistatin	NM_008046.2	AAGTGTATCACAAAGTCCTGTGAA<br>GGCGTATGTGGCATTGTC	162
Car14	Carbonic anhydrase 14	NM_011797.2	CACCTCTGCGTTCTCTTCCT<br>GAATGTGACTGTGGCCTGTG	125
LPAR4	Lysophosphatidic acid receptor 4	NM_175271.4	GAAGTGCGAGTTGCCAGTTT<br>GGAGGCAGACGATCAGAGAG	122
Gapdh*	Glyceraldehyde-3-phosphate dehydrogenase	NM_008084.2	AATGTGTCCGTCGTGGATCT<br>CCTGCTTCACCACCTTCTTG	79

*The reference gene used in the study.

Biology Open • Supplementary information

Table SM5 Bovine RT-qPCR primer sequences used in the study

Symbol	Description	GenBank Accession	Fwd and Rvs primer (5' - 3')	PCR product (bp)
SPIC	Spi-C transcription factor (Spi-1/PU.1 related)	NM_001076421.1	AGGGCTGAAGTGTCTTCCTG<br>ATGCTTGACCCAGCTTGTCT	80
GJB5	Gap junction protein, beta 5	NM_001205907.1	AAGCCCGAGGAAAAGAACAT<br>AAAGATGAGGTCGCCTGAGA	216
KLF4	Kruppel-like factor 4 (gut)	NM_001105385.1	CCCACACAGGTGAGAAACCTT<br>GTGTGGGTCACATCCACTGT	213
SCPEP1	serine carboxypeptidase 1	NM_001045909.2	GCTTGGATCTCTCCCATTGA<br>CCCCATCTGTGTTCTGTTCA	199
PSAP	Prosaposin	NM_174161.3	GGCTTCTGTGACGAGGTGAA<br>TCGCACACCTCGCAGTAAAT	152
TRIP6	Thyroid hormone receptor interactor 6	NM_001035469.1	GACGGCATTCCTTTCACTGT<br>CAATGTGAAAACTGCGATCC	166
JAKMIP2	Janus kinase and microtubule interacting protein 2	NM_001075897.2	AGAGGGAGGGGAAGAAATCCC<br>TGAATTCCTCTCAAGCAGGGG	250
DUSP6	Dual specificity phosphatase 6	NM_001046195.1	CCCATCTCGGATCACTGGAG<br>CACCAGGACGCCACAGTTTT	99
LIN28B	Lin-28 homolog B (C. elegans)	XM_002707838.3	CCCCCTCCAAAATGTTCCGA<br>CCCCCTCCAAAATGTTCCGA	108
SEMA6A	Sema domain, transmembrane domain (TM), and cytoplasmic domain, (semaphorin) 6A	NM_001193209.1	ATTGCCACGCTCTATGTCCT<br>AAAGCCAGCAGCTTCCTGTA	204
GAPDH*	POU domain, class 5, transcription factor 1	NM_001034034.2	TTCAACGGCACAGTCAAGG<br>ACATACTCAGCACCAGCATCAC	119

*The reference gene used in the study.

Biology Open • Supplementary information

Table SM6 Pig RT-qPCR primer sequences used in the study

Symbol	Description	GenBank Accession	Fwd and Rvs primer (5' - 3')	PCR product (bp)
POU5F1	POU domain, class 5, transcription factor 1	NM_001113060.1	GATCAAGCAGTGACTATTCGCA<br>GCTTCAGCAGCTTGGCAAAC	208
SPIC	Spi-C transcription factor (Spi-1/PU.1 related)	XM_003481736.2	CAGTCTCTGCAGAACATCCCT<br>TCTGGTCTATCCACTGAATAC	156
GJB5	Gap junction protein, beta 5	XM_003127741	TCTGGCTGTCCCTGGTCTT<br>CCGTGATCACCATGAAGAGA	512
KLF4	Kruppel-like factor 4 (gut)	GI2825099	CTCCTCTTCGTCGTCGCCGT<br>CAGCGACGCCTTCAGCACGA	521
SCPEP1	Serine carboxypeptidase 1	XM_005656968.1	ATGCCCACATGTTCTGGTG<br>CAAAGTTGCACTGGATGGTC	400
PSAP	Prosaposin	XM_005671042.1	GTGAAGACAGCATCCGACTG<br>CTTGCACGTGGCAGACATG	218
TRIP6	Thyroid hormone receptor interactor 6	XM_003354478.2	TTGTGAGAGCTGCTATGTGG<br>TGGTGACGGTGGCTGAGAG	421
JAKMIP2	Janus kinase and microtubule interacting protein 2	XM_003124075.4	AGAGCGCCTTAAGCTCTTAC<br>TCATCCAAAGCCTCCTTCTGCAG	296
DUSP6	Dual specificity phosphatase 6	NM_001267842.1	AGGAGTTCGGCATCAAGTAC<br>CAATATCCAGACAGACAGTGTG	272
LIN28B	Lin-28 homolog B (C. elegans)	NM_001123133.1	CAGAGTAAGCTGCACATGGAGG<br>GTAGGCTGGCTTTCCCTGTG	344
SEMA6A	Sema domain, transmembrane domain (TM), and cytoplasmic domain, (semaphorin) 6A	XM_005661593.1	CAGATACCGCCTGACCAAAA<br>CCCATGACAAAAGCCAGAAT	553
GAPDH*	Glyceraldehyde-3-phosphate dehydrogenase	NM_001206359.1	TCGGAGTGAACGGATTTG<br>CCTGGAAGATGGTGATGG	219

*The reference gene used in the study.
